# Alpha Mangostin and Cisplatin as Modulators of Exosomal Interaction of Ovarian Cancer Cell with Fibroblasts

**DOI:** 10.3390/ijms23168913

**Published:** 2022-08-10

**Authors:** Paulina Borzdziłowska, Ilona Bednarek

**Affiliations:** Department of Biotechnology and Genetic Engineering, Faculty of Pharmaceutical Sciences in Sosnowiec, Medical University of Silesia in Katowice, 40-055 Katowice, Poland

**Keywords:** exosomes, α-mangostin, cisplatin, ovarian cancer, metastasis, tumor microenvironment

## Abstract

The diversity of exosomes and their role in the microenvironment make them an important point of interest in the development of cancer. In our study, we evaluated the effect of exosomes derived from ovarian cancer cells on gene expression in fibroblasts, including genes involved in metastasis. We also attempted to evaluate the indirect effect of cisplatin and/or α-mangostin on metastasis. In this aspect, we verified the changes induced by the drugs we tested on vesicular transfer associated with the release of exosomes by cells. We isolated exosomes from ovarian cancer cells treated and untreated with drugs, and then normal human fibroblasts were treated with the isolated exosomes. Changes in the expression of genes involved in the metastasis process were then examined. In our study, we observed altered expression of genes involved in various steps of the metastasis process (including genes related to cell adhesion, genes related to the interaction with the extracellular matrix, the cell cycle, cell growth and proliferation, and apoptosis). We have shown that α-mangostin and/or cisplatin, as chemotherapeutic agents, not only directly affect tumor cells but may also indirectly (via exosomes) contribute to delaying metastasis development.

## 1. Introduction

A continuing concern is the noted fact that in highly developed countries ovarian cancer is one of the most common causes of death. Importantly, as many as 58% of cases are diagnosed as metastatic [1]. Laboratory screening of tumor antigens 125 (CA-125) with gynecological evaluation and transvaginal ultrasound is not sufficient for early disease detection.

There are four histological subtypes of epithelial ovarian cancer: serous, endometrioid, clear cell, and mucinous. Serum cancer is the most common and comes in two forms. The low-grade subtype (LGSC) accounts for approximately 10% of all serous subtypes. It shows minimal nuclear atypia and slight molecular changes, often involving KRAS and BRAF mutations. The high-grade subtype (HGSC) accounts for 90% of all serous subtypes. It is characterized by significant nuclear atypia and a high frequency of p53 and *BRCA 1/2* mutations in the absence of *KRAS/BRAF* [2,3].

Conventional treatments for ovarian cancer include a combination of chemotherapy and surgery. The extent of the surgical procedure depends on the stage of the cancer. It is necessary to consider laparoscopic surgery and perform a genetic risk assessment (mainly *BRCA 1/2* mutation). Basic and neoadjuvant chemotherapy is mainly based on the use of platinum or carboplatin in combination with paclitaxel [4]. The efforts to find effective drugs for use in maintenance therapy continue. So far, bevacizumab and PARP inhibitors are used most often [5,6]. The presence of resistance of ovarian cancer cells to platinum is also a challenge. Patients with platinum-resistant ovarian cancer relapse within six months of cytoreductive surgery and neoadjuvant chemotherapy [3].

One trend in the investigation of new drugs is the search for compounds of natural origin. A lot of research is conducted on compounds belonging to the xanthones group, and one of the most important representatives is α-mangostin. This compound comes from the tropical *Garcinia Mangostana* tree, whose purple fruit is rich in a wide variety of flavonoids and xanthones. Research on α-mangostin confirms its many properties, including antioxidant, antibacterial and antifungal [7]. It has also been shown to have broad anti-cancer activity at various stages of disease development by modulating various molecular signaling pathways involved in cancer development [8].

The exact molecular mechanism for the development of ovarian cancer is still unknown. Increasing attention has been paid to the study of the neoplastic microenvironment in recent years. It probably plays an essential role in the development and metastasis of ovarian cancer. A detailed description and explanation of the mechanisms may lead to the discovery of better potential diagnostic biomarkers and help in the development of a new and better therapy.

The tumor microenvironment is a term that includes: the extracellular matrix (ECM); stromal cells; immune cells (including macrophages, natural killer cells, T-cells (CD4+, CD8+, T-regulatory cells), dendritic cells and B-cells); hormones, cytokines, and other bioactive molecules released along the autocrine, endocrine, or paracrine pathways; ECM nanostructures and mechanical changes due to the movement of the body or body fluids [9]. One of the elements of the neoplastic microenvironment is fibroblasts. Cancer-associated fibroblasts (CAFs) have features similar to myofibroblasts. They are one of the most important elements of cancer cells. Features of CAF are similar to active fibroblasts, with the difference that in neoplastic tissue these fibroblasts remain constantly active. It is associated with the constant stimulation of cell metabolism, including the production of collagen and elastin, and the secretion of enzymes [10]. The activation process may occur under the influence of various stimuli, i.e., growth factors, direct cell–cell contact, or oxidative stress. The continuous excited state of fibroblasts in the neoplastic tissue promotes the proliferation and growth of tumor cells. Moreover, the activation state influences other molecular pathways that contribute to tumor growth [11]. In the ovarian cancer microenvironment outside of the stromal cells (cancer-associated fibroblasts, tumor-associated macrophages (TAM), T-regulatory cells (Treg), myeloid-derived suppressor cells (MDSCs), endothelial cells and pericytes), exosomes are also important components [12].

During the gradual development of the neoplastic process, the normal environment is transformed into a cancerous one [13]. The changes that occur in this process concern various elements of the microenvironment, both the ECM and the surrounding cells. Until now, there is no single theory regarding the formation and origin of CAF cells. Current research indicates the origin of neoplastic fibroblasts from various cells, including: tissue resident fibroblasts, mesenchymal stem cells, hematopoietic stem cells, epithelial cells or endothelial cells [14]. In ovarian cancer, the surrounding tumor cells that make up the stroma are an important element in the development of the tumor. In the process of tumor growth, cells that are part of the microenvironment change their morphological and molecular features. The stromal fibroblasts of ovarian cancer become activated and take on the characteristics of cancer-associated fibroblasts. The changes that occur in fibroblasts further contribute to the faster development of both primary tumors and metastases [13]. The process of converting normal fibroblasts into activated, cancerous ones is complex. An important role in this process is played by exosomes derived from neoplastic cells, which, as part of the microenvironment, transfer the “neoplastic load” to normal cells. As a consequence, they support the process of tumor development and the formation of metastases [15].

A complete definition of exosomes has been developed over the years, but, to date, there are no clear defining criteria. The diameter of an exosome depends mainly on its origin. The lipid membrane provides rigidity, and the smallest vesicle must be at least 30 nm in diameter and is dependent on the lipid bilayer. It has been calculated that the total “load” of exosomes probably cannot exceed 100 proteins and 10,000 nucleotides of nucleic acid [16]. Despite the diversity of exosomes, they share common features, and the most common methods used to identify them are: tetraspanins, Alix, flotillin, TSG101, and Rab5b [17]. An interesting aspect is the acquisition of chemo-resistance by cancer cells linked to CAF activity. There is an emerging need to develop combination therapy, where combinations of classical cytostatic would be used along with new modulators that increase not only the effectiveness of anti-cancer therapy but also regulate CAF activation.

Modulation of the neoplastic microenvironment through various drug combinations can significantly influence the development of metastases. We have noted that ovarian cancer cells of different stages secrete different numbers of exosomes depending on the treatment conditions [18]. It can be assumed that the cargo carried by such exosomes also varies. In this context, the change in expression of several genes involved in metastasis under the influence of exosomes of different origins was analyzed and evaluated. The aim of the study was to verify the effect of exosomes released by ovarian cancer cells (established cell lines: SKOV-3 cisplatin-resistant and TOV-G21 cisplatin-sensitive) on gene expression in normal fibroblasts. Cisplatin, which is used in the treatment of ovarian cancer, was chosen as the agent that affects tumor cells and thus indirectly affects the released exosomes, while α-mangostin was used as a potential modulator of classical chemotherapy. The research presented here aims to clarify the role of exosomes in modulating fibroblast activity as components of the ovarian cancer microenvironment. In addition, the goal is to find answers to the question of how the drug combination we used indirectly affects changes in exosomes and subsequently the potential reprogramming of fibroblasts in the tumor microenvironment.

## 2. Results

In the first stage of the study, the XTT cytotoxicity test was performed for α-mangostin and/or cisplatin, and the IC50 value was determined (Table 3). The exosomes were then isolated from untreated and drug-treated SKOV-3 and TOV-21G cells. The quantitative and qualitative analysis of the results was presented in the publication [18].

Normal NHDF fibroblasts were exposed to exosomes isolated from tumor cells. Then, after the RNA isolation, the changes in the expression of genes involved in the metastasis process were analyzed (using the Human Tumor Metastasis PCR Array (Qiagen, Hilden, Germany)). Table 1 shows the set of analyzed genes. The gene pool under study was divided into categories taking into account various aspects and complexity of the entire metastasis process.

### 2.1. Analysis of Gene Expression Changes Involved in Cell Adhesion

**Figure 1:**
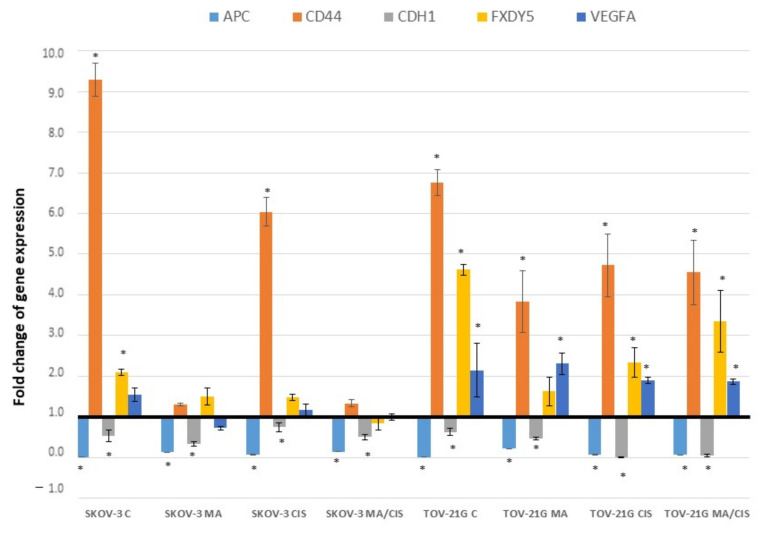
Effect of exosomes derived from SKOV-3 and TOV-21G cells (untreated with drugs, α-mangostin and/or cisplatin-treated) on the expression of cell adhesion markers in fibroblasts. The data are shown as mean ± SD of triplicate experiments, statistically significant results * (*p* < 0.05).

The most important changes in the expression of genes involved in cell adhesion were noted for the genes: *APC*, *CD44*, *CDH1*, *FXDY5* and *VEGF*. A summary of the changes in gene expression was presented in Figure 1.

After the treatment of fibroblasts with neoplastic exosomes, the expression of the *APC* (Adenomatous polyposis coli) gene was significantly reduced. Exosomes from untreated cells of both tested lines caused a decrease in *APC* gene expression by more than 99% (compared to untreated normal fibroblasts). On the other hand, treatment of fibroblasts with exosomes from SKOV-3 and TOV-21G cells treated with α-mangostin and/or cisplatin resulted in slight changes in the expression of this gene. The most significant differences were noted for exosomes released from cells treated with α-mangostin. Correspondingly, for exosomes derived from the SKOV-3 line by 87.8%, and for the TOV-21G line by 78.8%

Significant changes in the expression of the *CD44* gene (CD44 molecule—Indian blood group) were noted in the study. Under the influence of exosomes derived from untreated SKOV-3 and TOV-21G cells, a significant increase in the expression of this gene was observed, by more than 800% and 500%, respectively, as compared to normal fibroblasts. A significantly lower increase in *CD44* expression was observed in the treatment of fibroblasts with exosomes derived from ovarian cancer cells treated with α-mangostin and/or cisplatin. Treatment of normal fibroblasts with exosomes derived from SKOV-3 and TOV-21G cells treated with α-mangostin contributed to the smallest increase in the expression of this gene compared to fibroblasts treated with exosomes from untreated tumor cells (by 30.1% and 283.2%, respectively).

There were no statistically significant differences in gene expression: *CDH6* (Cadherin 6, type 2, K-cadherin, fetal kidney) and *CDH11* (Cadherin 11, type 2, OB-cadherin, osteoblast) and *FAT1* (FAT tumor suppressor homolog 1, Drosophila), *ITGA7* (Integrin, alpha 7) and *SYK* (Spleen tyrosine kinase).

Statistically significant, slight changes in the expression of the *CDH1* gene (Cadherin 1, type 1, E-cadherin epithelial) were observed in fibroblast cells after treatment with tumor-derived exosomes. In the case of exosomes derived from untreated or drug-treated SKOV-3 lineage, a decrease in *CDH1* expression from 26.0% to 67.05% was noted. In the case of exosomes from the untreated or drug-treated TOV-21G lineage, a decrease in cadherin 1 expression ranging from 37.42% to 98.99% was noted.

Analysis of the expression of the *FXYD5* gene (FXYD domain-containing ion transport regulator 5) showed, in most cases, an increase in the expression of this gene after treatment of fibroblasts with exosomes derived from ovarian cancer cells. The observed gene expression changes differed depending on whether the exosomes originated from SKOV-3 or TOV-21G cells. The noted increase in *FXYD5* expression after treatment of cells with exosomes from the untreated SKOV-3 line was 109.88%, while in the untreated TOV-21G line, it was 361.58%. The increase in *FXYD5* expression was less after treatment of fibroblasts with exosomes derived from drug-treated ovarian cancer cell lines tested (both SKOV-3 and TOV-21G).

Treatment of fibroblasts with exosomes from ovarian cancer cells changed the expression of the *VEGFA* gene (Vascular endothelial growth factor A). The changes observed were different depending on the origin of the exosomes. An increase in *VEGFRA* expression was observed after treatment with exosomes from untreated and cisplatin-treated SKOV-3 cells. In contrast, a decrease in expression was observed after treatment with exosomes from SKOV-3 cells treated with α-mangostin and a drug combination (α-mangostin and cisplatin). Exosomes isolated from TOV-21G cells (regardless of cell treatment conditions) contributed to the increase in the expression of the *VEGFRA* gene. The recorded increase in expression ranged from 88.39% to 130.15% (for untreated normal fibroblasts).

The studies evaluated the change in expression of four selected transmembrane receptors: *CD44*, *ITGA7* (Integrin, alpha 7), *ITGB3* (Integrin, beta 3, platelet glycoprotein IIIa, antigen CD61), and *RPSA* (Ribosomal protein SA). Among the studied genes, significant changes in expression were noted for the *CD44* (description above) and *RPSA* genes. After treatment of fibroblasts with exosomes from untreated SKOV-3 cells, there was a decrease in *RPSA* gene expression by 30.19% (*p* < 0.05). After treatment of the fibroblasts with exosomes derived from the treated SKOV-3 cells, the decrease in expression was twice as high, at 83.91%, 60.77% and 62.23%, respectively, for SKOV-3 cells after treatment with α-mangostin, cisplatin, and drug combination.

Among the other genes involved in cell adhesion in the process of metastasis, the expression change of the following genes was assessed: *CTNNA1* (Catenin, cadherin-associated protein, alpha 1, 102kDa), *FN1* (Fibronectin 1), *MCAM* (Melanoma cell adhesion molecule), *MGAT5* (Mannosyl (alpha-1,6-)-glycoprotein beta-1,6-N-acetyl-glucosaminyltransferase), *MTSS1* (Metastasis suppressor 1). No statistically significant changes in the expression in fibroblasts after treatment with exosomes were observed for any of the above genes.

### 2.2. Analysis of Gene Expression Changes Related to the Extracellular Matrix

**Figure 2:**
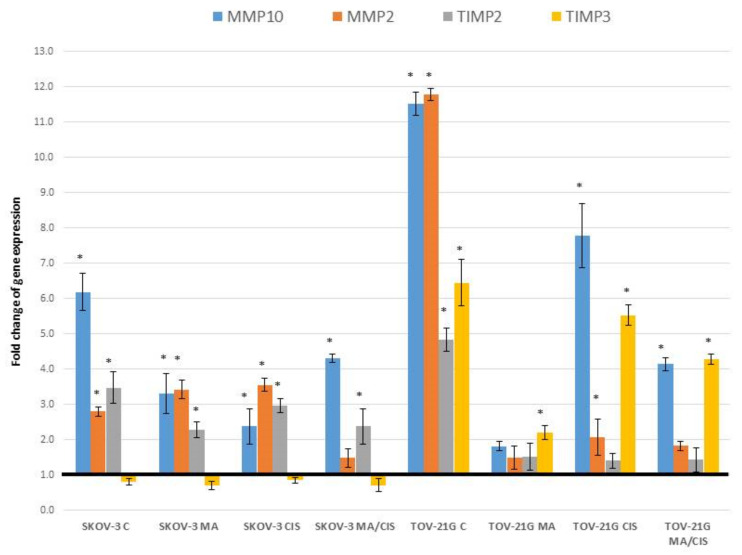
Effect of exosomes derived from SKOV-3 and TOV-21G cells (untreated with drugs, α-mangostin and/or cisplatin-treated) on the expression of extracellular matrix markers in fibroblasts. The data are shown as mean ± SD of triplicate experiments, statistically significant results * (*p* < 0.05).

The expression change of 13 genes related to ECM remodeling was analyzed. From among the tested genes, statistically significant differences were noted for four genes: *MMP2*, *MMP10*, *TIMP2* and *TIMP3*. The results are shown in Figure 2.

The studies showed a change in expression for two of the seven analyzed *MMP* (Matrix metallopeptidase) genes. In fibroblasts treated with exosomes from untreated ovarian cancer cells, a more significant increase in *MMP2* and *MMP10* expression was observed for the exosomes derived from the TOV-21G lineage. Exosomes derived from the untreated SKOV-3 lineage increased the expression of *MMP10* by 518.37%. Treatment of SKOV-3 cells with both α-mangostin and cisplatin was associated with a lower increase in this gene expression in the case of α-mangostin by 229.48%, cisplatin by 137.07%, and for both drugs by 330.45%.

On the other hand, the exosomes from the untreated TOV-21G line increased the expression of *MMP10* in fibroblasts by 1051.65%. The smallest increase in the expression of this gene was noted under the influence of exosomes derived from TOV-21G cells treated with α-mangostin (by 81.09%). Similarly, significant changes in gene expression were observed for the *MMP2* gene. Exosomes from untreated TOV-21G cells increased MMP2 expression by 1078.63%. The smallest increase in this gene was recorded for the treatment of fibroblasts with exosomes from TOV-21G cells treated with α-mangostin by 48.61%. Much smaller differences in *MMP2* expression were noted when treating fibroblasts with exosomes from SKOV-3 cells. Exosomes from untreated cells increased expression by 179.59%. Exosomes from α-mangostin and cisplatin-treated SKOV-3 cells contributed to an increase in expression by 241.91% and 254.42%, respectively. The smallest increase in the expression of this gene in fibroblasts was observed under the influence of exosomes from SKOV-3 cells treated simultaneously with α-mangostin and cisplatin.

Comparing changes in the expression of *TIMP* genes (TIMP metallopeptidase inhibitor), significant changes were noted in the case of *TIMP2* and *TIMP3*. There was no change in *TIMP4* gene expression. Exosomes derived from SKOV-3 ovarian cancer cells altered the expression of only the *TIMP2* gene. The highest increase in expression in fibroblasts was noted for exosomes derived from untreated cells (by 246.36%). In the case of exosomes from the treated SKOV-3 cells, an increase in *TIMP2* expression was noted, respectively, for α-mangostin by 128.27%, and for cisplatin by 196.11%, and for treatment with two drugs by 137.30%. TOV-21G exosomes altered both *TIMP2* and *TIMP3* expression. The observed differences in expression of the *TIMP2* gene in fibroblasts were similar to treatment with exosomes from SKOV-3 cells. Under the influence of untreated exosomes from TOV-21G cells, an increase in *TIMP2* expression by 382.59% was noted. In the remaining cases, the increase in the expression of this gene was comparable, ranging between 40.25% and 51.47%. The greatest increase in TIMP3 expression was also noted in exosomes from untreated TOV-21G cells (by 543.83%).

There were no statistically significant changes in gene expression: *COL4A2* (Collagen, type IV, alpha 2), *HPSE* (Heparanase) and *SERPINE1* (PAI-1, Serpin peptidase inhibitor, clade E, nexin, plasminogen activator inhibitor type 1, member 1).

### 2.3. Analysis of Gene Expression Changes Related to the Cell Cycle

In the analysis of changes in the expression of genes involved in regulating the cell cycle in fibroblasts under the influence of exosomes from ovarian cancer cells, significant changes were found for five genes. No changes were noted for the genes: *CDKN2A* (P16INK4A, Cyclin-dependent kinase inhibitor 2A, melanoma, p16, inhibits CDK4), *IL1B* (Interleukin 1, beta), *KRAS* (V-Ki-ras2 Kirsten rat sarcoma viral oncogene homolog), *MTSS1* (Metastasis suppressor 1), *NF2* (Neurofibromin 2, merlin), *NME1* (NM23, Non-metastatic cells 1, protein), *PTEN* (Phosphatase and tensin homolog), *RB1* (Retinoblastoma 1), *TGFB1* (Transforming growth factor, beta 1) and *MYC* (V-myc myelocytomatosis viral oncogene homolog (avian)).

**Figure 3:**
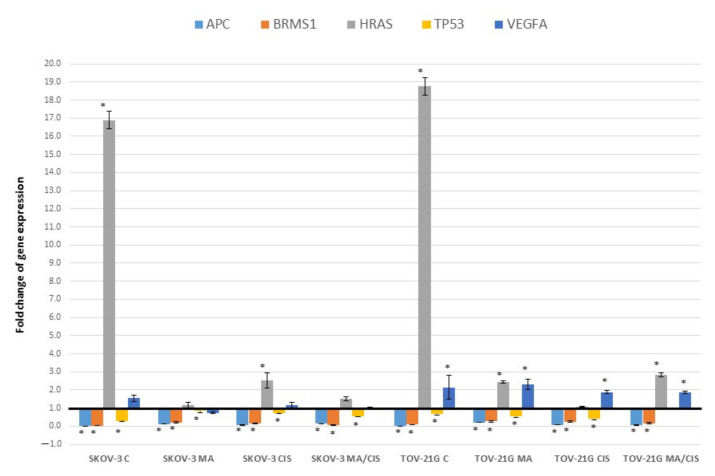
Effect of exosomes derived from SKOV-3 and TOV-21G cells (untreated with drugs, α-mangostin and/or cisplatin-treated) on the expression of cell cycle markers in fibroblasts. The data are shown as mean ± SD of triplicate experiments, statistically significant results * (*p* < 0.05).

Figure 3 shows changes in the expression of genes involved in the cell cycle for which statistically significant differences were noted. Changes in *APC* and *VEGFA* gene expression were described above (Section 2.1).

In our study, we observed a decrease in the expression of the *BRMS1* gene (Breast cancer metastasis suppressor 1) in fibroblasts after treatment with exosomes of tumor origin. There were no differences between the origins of the SKOV-3 and TOV-21G exosomes. Slight differences in the decrease in expression of this gene were observed depending on the origin of the exosomes from untreated or drug-treated tumor cells. The observed decrease in *BRMS1* expression was between 72.59% and 96.37%.

The observed differences in expression of the *HRAS* gene (V-Ha-ras Harvey rat sarcoma viral oncogene homolog) were significant depending on whether the fibroblasts were treated with exosomes derived from untreated or drug-treated ovarian cancer cells. In the case of exosomes derived from untreated SKOV-3 cells, an increase in the expression of *HRAS* in fibroblasts of over 1500% was observed, while in the case of exosomes from untreated TOV-21G cells it was over 1700%. Treatment of ovarian cancer cells with α-mangostin and/or cisplatin also increased the expression of this gene in fibroblasts under the influence of exosomes. However, the increase was significantly smaller, between 8.41% and 183.73%.

Changes in the expression of *TP53* (Tumor protein p53) in fibroblasts were different depending on the origin of the exosomes being treated. There was a 71.28% decrease in expression for exosomes from untreated SKOV-3 cells. The smallest decrease in *TP53* expression in fibroblasts was observed after treatment with exosomes from α-mangostin-treated SKOV-3 cells (by 16.22%). TOV-21G exosomes did not show such large differences in *TP53* expression. There was a 33.96% decrease in *TP53* expression in fibroblasts by exosomes derived from untreated TOV-21G cells.

### 2.4. Analysis of Gene Expression Changes Related to Cell Growth and Proliferation

The change in the expression of genes involved in the proliferation process was analyzed under the influence of exosomes derived from SKOV-3 and TOV-21G cells. Table 1 provides a list of the studied genes. Among the genes involved in the regulation of cell proliferation, no expression changes were noted for the following genes: *CDKN2A*, *CTBP1* (C-terminal binding protein 1), *GNRH1* (Gonadotropin-releasing hormone 1, luteinizing-releasing hormone), *IL1B* (Interleukin 1, beta), *NF2*, *NME1*, *SSTR2* (Somatostatin receptor 2), *IGF1* (Insulin-like growth factor 1, somatomedin C) and *TSHR* (Thyroid stimulating hormone receptor).

**Figure 4:**
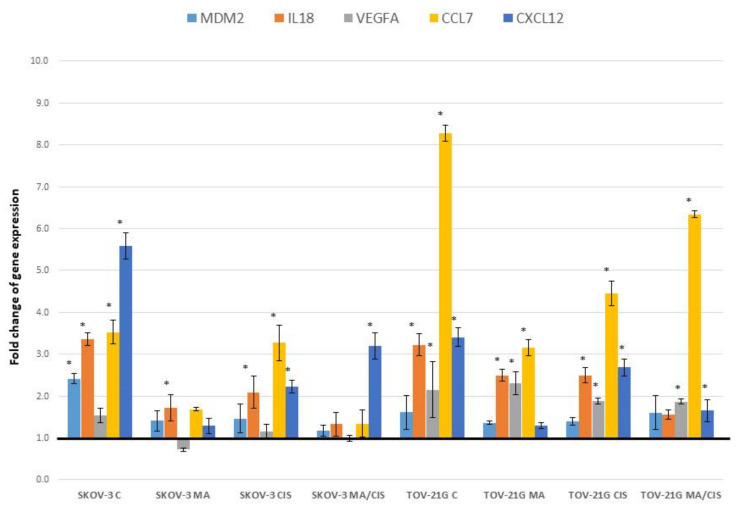
Effect of exosomes derived from SKOV-3 and TOV-21G cells (untreated with drugs, α- mangostin and/or cisplatin-treated) on the expression of cell growth and proliferation markers in fibroblasts. The data are shown as mean ± SD of triplicate experiments, statistically significant results * (*p* < 0.05).

Figure 4 shows changes in gene expression that have been statistically significant in the studies.

Analysis of changes in *MDM2* gene expression (Mdm2 p53 binding protein homolog, mouse) showed an increase in fibroblasts expression after treatment with exosomes derived from both SKOV-3 and TOV-21G cells. The observed increase in *MDM2* expression was lower for exosomes derived from α-mangostin and/or cisplatin-treated ovarian cancer cells.

Significant changes in expression were observed in the analysis of the *IL18* gene (Interleukin 18, interferon-gamma-inducing factor). The highest increase in expression was observed for cells treated with exosomes from untreated SKOV-3 and TOV-21G cells, by 235.74% and 222.51%, respectively. Exosomes from both studied ovarian cancer lines treated simultaneously with α-mangostin and cisplatin contributed to the smallest increase in *IL18* expression. The observed increase in *IL18* expression after treatment of fibroblasts with exosomes from α-mangostin or cisplatin-treated cells ranged from 72.06% to 149.71%.

Changes in *VEGFA* gene expression are described above (Section 2.1).

Among the growth factors, hormones and cytokines important in the metastasis process, significant changes in the expression in fibroblasts after exosome treatment were observed for the genes: *VEGFA*, *IL18*, *CCL7* (MCP-3, Chemokine (C-C motif) ligand 7) and *CXCL12* (SDF1, Chemokine (C-X-C motif) ligand 12). There were no differences for the genes: *GNRH1*, *HGF* (Hepatocyte growth factor, hepapoietin A; scatter factor), *IGF1*, *TGFB1*, *IL1B* and *TNFSF10* (TRAI, Tumor necrosis factor (ligand) superfamily, member 10).

The changes we observed in the expression of the *CCL7* gene (MCP-3, Chemokine (C-C motif) ligand 7) were greater after treating fibroblasts with exosomes derived from TOV-21G cells than from SKOV-3 cells. Exosomes derived from untreated TOV-21G cells increased the expression of *CCL7* in fibroblasts by 727.94%. In the case of exosomes derived from drug-treated TOV-21G cells, the observed increase in expression was lower. For the treatment of cells with α-mangostin, it was 216.20%, cisplatin 345.70%, and with both drugs, 534.28%. The expression of *CCL7* in fibroblasts after treatment with exosomes from SKOV-3 cells increased by 252.36%. After treatment with exosomes from drug-treated SKOV-3 cells, the increase was lower, between 34.13% and 226.99%.

The second chemokine for which we noted significant differences in expression is *CXCL12* (SDF1, Chemokine (C-X-C motif) ligand 12). Exosomes from both examined ovarian cancer lines contributed to the increase in the expression of *CXCL12* in fibroblasts. The observed increase for exosomes derived from untreated SKOV-3 cells was 458.65%, while for TOV-21G cells it was 240.47%. For the exosomes of both studied cell lines, after treatment with α-mangostin and cisplatin simultaneously, a two-fold lower increase in the expression of the *CXCL12* gene was observed (in relation to the treatment of fibroblasts with exosomes from untreated ovarian cancer cells). In the case of exosomes from the SKOV-3 line, the increase was 219.99%, and for exosomes from TOV-21G cells, it was 65.49%.

**Figure 5:**
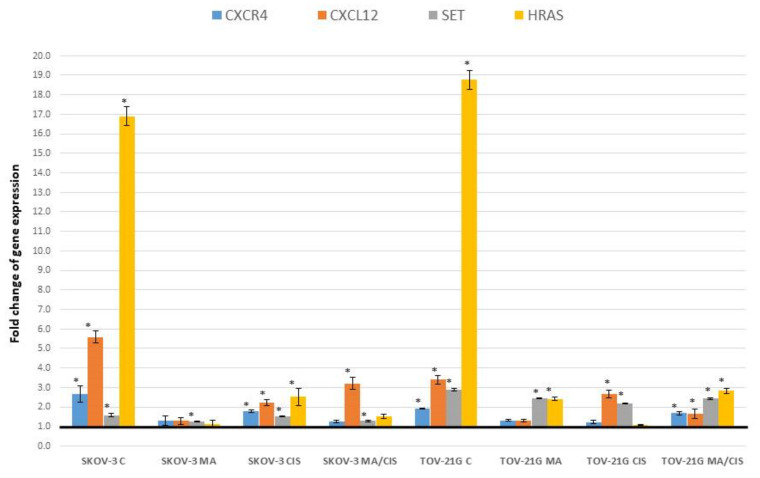
Effect of exosomes derived from SKOV-3 and TOV-21G cells (untreated with drugs, α- mangostin and/or cisplatin-treated) on the expression of receptors CXCR4 and their ligand CXCXL12 and SET and HRAS gene in fibroblasts. The data are shown as mean ± SD of triplicate experiments, statistically significant results * (*p* < 0.05).

Analyzing changes in the expression of genes important for the metastasis process in cell growth and proliferation, we found changes for only one receptor *CXCR4* (Chemokine (C-X-C motif) receptor 4). Figure 5 shows the expression changes of these receptors together with their corresponding ligands.

Fibroblasts treated with exosomes from untreated SKOV-3 cells increased the expression of the *CXCR4* gene by 165.75%. Exomes from SKOV-3 cells treated simultaneously with α-mangostin and cisplatin contributed to the smallest increase in the expression of this gene in fibroblasts (by 23.93%). The observed differences were smaller for TOV-21G-derived exosomes. Fibroblasts treated with exosomes from untreated TOV-21G cells increased the expression of *CXCR4* by 92.08%. The smallest increase of this gene was noted under the influence of exosomes derived from TOV-21G cells treated with cisplatin (by 22.70%).

There were no changes for the following genes among cell receptors involved in cell growth and proliferation: *CXCR2* (Chemokine (C-X-C motif) receptor 2), *EPHB2* (EPH receptor B2), *FLT4* (Fms-related tyrosine kinase 4), *KISS1R* (KISS1 receptor), *MET* (Met proto-oncogene, hepatocyte growth factor receptor), *NR4A3* (NOR1, Nuclear receptor subfamily 4, group A, member 3), *PLAUR* (UPAR, Plasminogen activator, urokinase receptor), *RORB* (RAR-related orphan receptor B), *SSTR2* and *TSHR*.

Change in the expression of other genes involved in cell growth that may play an important role in the metastasis process was also assessed. Significant changes in gene expression in fibroblasts after treatment with exosomes from ovarian cancer cells were observed for *HRAS* and *SET* (SET nuclear oncogene). Figure 5 shows the expression changes of these genes. Changes in the expression of the *HRAS* gene were described in Section 2.3.

The increase in expression that we noted for the *SET* gene was three times greater after treatment with exosomes derived from TOV-21G cells compared to exosomes derived from SKOV-3 cells. The highest increase in *SET* expression was observed for exosomes from untreated cells of both ovarian cancer lines (by 58.26% for exosomes from SKOV-3 line and 188.20% for TOV-21G cells). Treatment of SKOV-3 and TOV-21G cells with α-mangostin and/or cisplatin resulted in a smaller increase in SET expression (relative to exosomes derived from untreated ovarian cancer cells).

Among the additional genes involved in cell growth, no significant changes were noted for the genes: *DENR* (Density-regulated protein), *EWSR1* (Ewing sarcoma breakpoint region 1), *MYC*, *SRC* (V-src sarcoma (Schmidt-Ruppin A-2) viral oncogene homolog, avian), *SYK* (Spleen tyrosine kinase) and *TRPM1* (Transient receptor potential cation channel, subfamily M, member 1).

### 2.5. Analysis of Gene Changes Related to the Process of Apoptosis Expression

In our study, we assessed eight genes involved in apoptosis, which also play an important role in the metastatic process. For the following genes, we found no significant changes in expression in fibroblasts after treatment with exosomes from ovarian cancer cells: *HTATIP2* (HIV-1 Tat interactive protein 2, 30 kDa), *IL1B*, *TGFB1* and *TNFSF10* (TRAIL, Tumor necrosis factor (ligand) superfamily, member 10).

**Figure 6:**
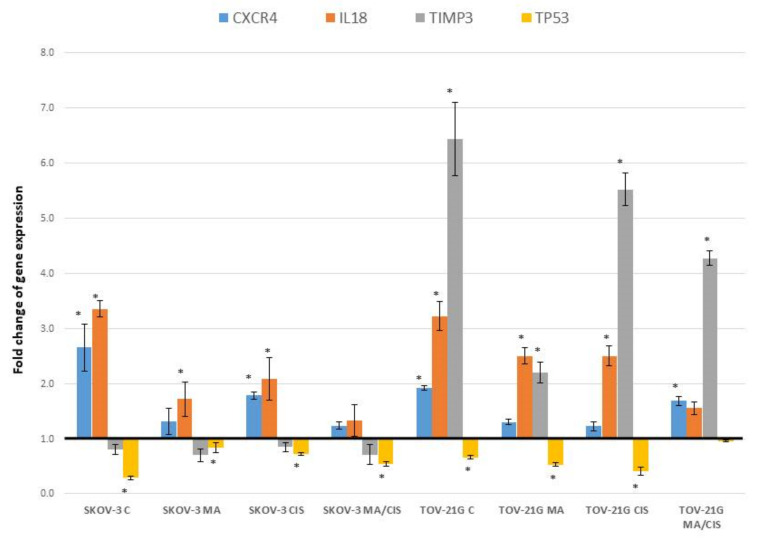
Effect of exosomes derived from SKOV-3 and TOV-21G cells (untreated with drugs, α-mangostin and/or cisplatin-treated) on the expression of apoptosis markers in fibroblasts. The data are shown as mean ± SD of triplicate experiments, statistically significant results * (*p* < 0.05).

Treatment of fibroblasts with exosomes derived from ovarian cancer cells changed the expression of the following genes involved in the apoptosis process: *CXCR4*, *IL18*, *TIMP3* and *TP53*. Changes in the expression of these genes are described above in Section 2.2, Section 2.3 and Section 2.4. Figure 6 summarizes the changes we observed for genes that play an important role in apoptosis in the metastatic process.

### 2.6. Analysis of Changes in the Expression of Transcription Factors Involved in the Metastasis Process

Changes in the expression of 14 genes of transcription factors that play an important role in metastasis were analyzed. The analyzed genes included: *CHD4* (Chromodomain helicase DNA binding protein 4), *ETV4* (Ets variant 4), *EWSR1*, *HTATIP2*, *MTA1* (Metastasis associated 1), *MYC*, *MYCL* (V-myc myelocytomatosis viral oncogene homolog 1, lung carcinoma derived, avian), *NR4A3*, *RB1*, *RORB*, *SMAD2* (MADH2, SMAD family member 2), *SMAD4* (MADH4, SMAD family member 4), *TCF20* (Transcription factor 20, AR1) and *TP53*. In fibroblasts after treatment with exosomes derived from ovarian cancer cells, statistically significant changes were observed only for *TP53* as mentioned previously in Section 2.3.

### 2.7. Analysis of Changes in the Expression of Additional Genes Associated with the Metastasis Process

In the conducted research, seven additional genes were selected and assigned a role in the metastasis process. The expression change of the following genes was analyzed *CD82* (CD82 molecule), *CST7* (Cystatin F, leukocystatin), *CTSK* (Cathepsin K), *CTSL* (Cathepsin L1), *KISS1* (KiSS-1 metastasis-suppressor), *METAP2* (Methionyl aminopeptidase 2) and *NME4* (Non-metastatic cells 4, protein expressed in) in fibroblasts after treatment with exosomes from ovarian cancer cells. For none of the analyzed genes, no statistically significant differences were found.

### 2.8. Summary of the Obtained Results

Table 2 lists the changes in gene expression level for all genes that show the statistical significance which are described in the above sections.

## 3. Discussion

Ovarian cancer is a heterogeneous neoplasm and the development of metastases is not well understood yet [19]. It is known that one aspect that strongly contributes to the faster development of metastasis is the changes in the stromal fibroblasts of ovarian cancer. Tumor lesions are not just cancer cells. Tumors create a complex microenvironment where normal cells such as fibroblasts, immune cells, vascular endothelial cells, adipocytes and others are found alongside cancer cells. The vast majority of this so-called tumor microenvironment is made up of fibroblasts. So-called resting fibroblasts in the tumor microenvironment under the influence of the paracrine activity of cancer cells undergo phenotypic reprogramming becoming cancer-associated fibroblasts (CAFs) recruited by the tumor [15]. CAFs are attributed to a number of properties associated with cancer progression. Interactions in the tumor microenvironment are known to be bidirectional, and exosomes are the main carrier of information passed between cells. Failures in chemotherapy or radiotherapy are largely associated with the activity of cells in the microenvironment, including fibroblasts. Consequently, new therapeutic approaches have emerged, where it is assumed, among other things, either to block cytokines released by CAFs or to prevent/reverse the phenotypic reprogramming of resting fibroblasts in CAFs. In this light, it seems important to describe changes in gene expression in resting fibroblast cells under the influence of factors carried by exosomes secreted by tumor cells.

The undertaken studies focused on the analysis of changes in the expression of genes in normal fibroblasts, where exosomes of different origins were the modulatory factor. Being one of the important elements of the microenvironment, including the tumor microenvironment, exosomes contribute to the development of metastasis. The molecular cargo carried by exosomes from cancer cells to normal cells contributes to metastasis in new locations [20]. In the present study, we focused on analyzing changes in the expression of genes involved in the metastatic process. We assessed the change in expression of genes involved in cell adhesion, extracellular matrix, cell cycle, cell growth and proliferation, apoptosis, and expression of transcription factor genes and regulators relevant to this process.

Two cell lines were selected for research: cisplatin-resistant SKOV-3 and TOV-21G (grade 3, stage III, primary malignant adenocarcinoma; clear cell carcinoma). Cancer cells were treated with two drugs: α-mangostin (a natural plant derivative) and cisplatin (a currently used chemotherapeutic agent). In addition, an attempt has been made to evaluate combinations of these drugs in the treatment of ovarian cancer. Earlier studies have shown that in the range of certain concentrations, α-mangostin with cisplatin shows a synergistic effect. It is possible to achieve the same cytotoxic effect for a drug combination with a lower concentration of cisplatin as compared to the use of cisplatin alone. These results give hope that the reduction in the concentration of cisplatin in combination with α-mangostin will be associated with a reduction in numerous side effects in relation to the currently used cisplatin alone [18,21,22]. The use of new drugs in the treatment of cancer may be more than just reducing the side effects of chemotherapy. An important aspect is the influence of drugs on the cancer cell’s microenvironment. Our studies to date have shown that treatment of SKOV-3 and TOV-21G cells with α-mangostin and/or cisplatin leads to changes in the number of exosomes released. We also showed that these exosomes, depending on the origin and treatment conditions, show different activity against normal fibroblasts [18]. In this study, we attempted to assess the change in the expression of genes involved in metastasis in normal fibroblasts under the influence of neoplastic exosomes.

Adhesive molecules are known to play an essential role in cell-to-cell interactions as well as in interactions between the cell and the surrounding environment. One aspect of the complex process of metastasis is the adhesion capacity of cells. Regulation of the Wnt/β-catenin pathway plays an important role in many cancers as well as in ovarian cancer. It is known that abnormalities in the Wnt pathway are associated with the maintenance of CSC cells (tumor stem cells), promotion of metastasis, increased survival of cancer cells and chemoresistance [23,24]. The analysis of the *APC* gene expression, which is one of the elements of the Wnt pathway, showed that exosomes derived from ovarian cancer cells lead to a significant reduction in the expression of this gene in normal fibroblasts. It was noted that the decrease was slightly smaller in the exosomes derived from the α-mangostin treated tumor cells. The decrease in APC protein concentration or its mutations in neoplastic cells is associated with an increase in β-catenin, which leads to activation of target genes of the Wnt pathway [25]. The studies showed that normal cells treated with exosomes from untreated tumor cells had the lowest expression of the *APC* gene. Exosomes derived from α-mangostin and/or cisplatin treatment also contribute to the decrease in *APC* gene expression, but not to such a great degree. It is known that the load carried by exosomes is variable [26]. Given that APC affects numerous chemoresistant pathways, altering responses to different classes of chemotherapeutic agents through tumor microenvironment-derived mechanisms our observations of altered *APC* expression in fibroblasts treated with exosomes harvested from a population of modulated ovarian cancer cells seem particularly relevant.

The second important cell adhesion molecule involved in metastasis is the *CD44* protein. It is a cell membrane molecule that is involved in the adhesion and cell-to-cell and cell-to-matrix interactions. The *CD44* adhesive molecule has many ligands, such as: hyaluronan, chondroitin, collagen, laminin and fibronectin. In addition, it binds to hyaluronic acid (HA), which increases the aggregation of macrophages, lymphocytes and fibroblasts. On the other hand, *CD44* by combining with hyaluronan contributes to its degradation, which may lead to morphological changes in the tissue. The adhesion of neoplastic cells becomes more efficient when it has more CD44 protein on their surface, which by binding with hyaluronic acid affects the growth and development of the tumor [27]. In our research, we showed that exosomes derived from ovarian cancer cells lead to an increase in the expression of the *CD44* gene in stimulated fibroblasts. There was a lower increase in expression in fibroblasts following treatment with exosomes derived from α-mangostin and/or cisplatin-treated SKOV-3 and TOV-21G cells. The research described in the literature shows that exosomes secreted from highly metastatic ovarian cancer cells can promote migration and invasion in less metastatic ovarian cancer cells, which is associated with the expression of the *CD44* gene [28,29]. The observed lower increase in *CD44* expression in fibroblasts after treatment with exosomes from drug-treated tumor cells could potentially indicate a reduction in metastatic activity. This may be related to the slower development of new metastatic lesions.

The studies indicate the important role of E-cadherin in the process of cell adhesion. It has been shown that mutations or a decrease in the expression of the *CDH1* gene (Cadherin 1, epithelial) were associated with decreased intercellular adhesion. A characteristic feature of cells in the process of invasion and metastasis is the loss of normal adhesive function [30]. In our research, we found a decrease in the expression of the gene encoding E-cadherin after treating fibroblasts with exosomes of neoplastic origin. The observed decrease in expression varied depending on the origin of the exosomes. E-cadherin-mediated inactivation of the adhesion system is believed to be an essential element of cancer invasion and metastasis development. The relationship between *CDH1* and *FXYD5* expression is worth noting. In our research, we noted differences in the expression of the *FXYD5* gene, which encodes dysadherin. Dysadherin is a surface molecule. Research indicates that *FXYD5* overexpression is directly associated with an increased risk of metastasis. Its increased expression has been shown in many different types of cancer, such as gastric, colon, pancreatic and breast cancers [31]. Its expression is probably not the initiator of metastasis formation, but a prognostic factor driving its formation [32]. Overexpression of dysadherin leads to reduced cell-to-cell adhesion via the E-cadherin-dependent and independent pathways. The increased activity of dysadherin contributes to the reduction of the amount of e-cadherin, which results in morphological changes in the cell facilitating the development of a new tumor. It also changes the organization of actin and affects cell mobility [33]. In our study, we noted a decrease in the expression of the e-cadherin gene along with an increased expression of dysadherin in fibroblasts after treatment with exosomes released from ovarian cancer cells. The observed increase in *FXDY5* was higher when treating fibroblasts with exosomes derived from the untreated or drug-treated TOV-21G line compared to exosomes derived from the SKOV-3 line. In studies on breast cancer cells, the relationship between dysadherin and the activity of the CC chemokine (*CCL2*) has been described, which has a chemotactic effect on monocytes, T lymphocytes and NK cells. Additionally, it stimulates angiogenesis and cell migration in cancer cells [33]. Interestingly, it has also been shown in ovarian cancer cells that lowered *FXDY5* expression may not only be associated with decreased cell migration and adhesion but also sensitize cells to chemotherapeutic agents such as cisplatin [34,35]. In the context of our research, this may be of great importance. The load of exosomes from drug-treated ovarian cancer cells contributed to a smaller increase in dysadherin expression in normal fibroblasts. This may be associated with a higher sensitivity to chemotherapeutic agents of the newly formed metastatic focus.

Another important gene related to adhesion in the metastatic process, for which we have noticed significant changes in expression, is the *VEGFA* gene (Vascular endothelial growth factor A). Vascular endothelial growth factor is responsible for the formation of new vascular systems in and around tumors. A characteristic feature is that the vascular systems formed under the influence of *VEGFA* overexpression are lacking integrity. It means that the surrounding tissues are still hypoxic. Consequently, it is still associated with the overexpression of *VEGFA* in tumor tissues [36]. In our research, the treatment of fibroblasts with exosomes derived from TOV-21G cells contributed to an increase in the expression of the *VEGFA* gene. It is known that increased expression of this gene is associated with the activation of chemotaxis and activators of plasminogen and collagenase. It facilitates the process of growth and remodeling of blood vessels and provides endothelial cells with stimuli for survival. In neoplastic tissues, *VEGFA* also activates VE-cadherin, *MMP2* and *MMP9*. This translates into the stimulation of the entire process related to the formation of new blood vessels in the closeness of the cancerous tissues [37,38]. Neoplastic exosomes carrying the load and stimulating the growth of *VEGFA* expression in new cells may contribute to faster development of the entire microenvironment of the newly formed metastatic focus. Overexpression of *VEGFRA* in the secondary focus is associated with faster development of metastatic tissues, in which the entire vascular system develops, ensuring the survival of neoplastic tissues. In addition, the currently available studies also confirm that in ovarian cancer, *VEGFA* expression is associated with the development of chemoresistance of cancer cells and induction of autophagy [39].

The extracellular matrix plays an important role in the progression and development of metastatic foci. The family of metalloproteinases (MMPs) is responsible for the reconstruction of the ECM and, in the case of tumors, for the faster development of metastases. Among matrix metalloproteinases, there are: matrilisins, collagenases, stromelysins, gelatinases, membrane metalloproteinases and others. The activity of *MMPs* is regulated by *TIMPs*, endogenous inhibitors. There are four types of metalloproteinase inhibitors, and each has a different range of action and reacts with a different metalloproteinase [40,41]. Many studies indicate the involvement of *MMPs* in ovarian cancer as well [42]. In our research, we noted an increase in *MMP2* and *MMP10* expression under the influence of exosomes derived from ovarian cancer cells. Metalloproteinase 2 is one of the most widely described in the development of ovarian cancer and plays a very important role in the early stages of adhesion and metastasis [43]. Increased expression of *MMP2* was confirmed in both benign and malignant ovarian cancer cells [42]. In our research, we noted an increase in *MMP2* expression in fibroblasts under the influence of exosomes derived from SKOV-3 and TOV-21G cells. The mechanism of action of *MMP2* includes, inter alia, the cleavage of fibronectin and vitronectin (ECM proteins) into smaller pieces. The observed increase in the expression of this gene in normal fibroblasts may contribute to the faster development of a new metastatic focus [43]. The exact role of *MMP10* is unknown; however, it is known to play a significant role in vascular remodeling [44]. There is little research into the role of *MMP10* in ovarian cancer, but it has been reported to be upregulated in this tumor as well, and it is known to be associated with cancer progression [42]. In our study, we noted an increase in *MMP10* expression in fibroblasts following treatment with exosomes derived from ovarian cancer cells. The most important observation in the research is that exosomes derived from drug-treated tumor cells contribute to a smaller increase in the expression of *MMP2* and *MMP10*. This could potentially translate into slower development of metastasis.

In our study, we also noted an increase in the expression of *TIMP2* and *3* in fibroblasts after treatment with exosomes isolated from ovarian cancer cells. In the case of exosomes derived from SKOV-3 cells, we observed a change in expression only for *TIMP2*, while in the case of TOV-21G cells for *TIMP2* and *3*. The role of *TIMP* is not limited to the inhibition of *MMPs*. Studies show that *TIMPs* regulate a number of other MMP-independent signaling pathways, including the mitogen-activated protein kinase pathway (MAPK), protein kinase A cyclic adenosine monophosphate (cAMP), and activation of RAS pathways [40]. The role of *TIMP2* reported in the literature is associated with both the inhibition and initiation of *MMP2*. The role of *TIMP2* in the regulation of proliferation, invasion and chemoresistance in ovarian cancer cells is also attributed. The potential use of *TIMP2* as a therapeutic target in chemoresistant ovarian cancer is also considered [45]. In our research, exosomes derived from the cisplatin-resistant SKOV-3 line contributed to the increase in *TIMP2* expression in fibroblasts. Based on the available knowledge and the research results obtained by us, it is possible to assume and try to extend the scope of research to search for new therapeutic drugs in cisplatin-resistant ovarian cancer, where the therapeutic target will be *TIMP2*. The increase in the expression of both *MMP* and *TIMP* observed in the research may indicate the TIMP-independent regulation of MMPs, which is already described in the literature [45,46,47].

Disturbances in the regulation of the cell cycle are an important cause of the development of malignant neoplasms. Mutations or changes in the expression of genes involved in the signaling pathways that control the cell cycle are also the cause of the development of ovarian cancer [48]. In our study, we noted changes in the expression of five genes involved in the cell cycle in fibroblasts treated with exosomes derived from ovarian cancer cells. The p53 protein plays a key role in the maintenance of cellular homeostasis. Most of the high-grade serous ovarian carcinomas (HGSOCs) have an inactive *TP53* gene, mainly due to genetic mutations [49]. It was also confirmed that the presence of null mutations in the *TP53* gene is associated with the faster development of distant metastases in ovarian cancer. In our research, we noted a decrease in the expression of the *TP53* gene in fibroblasts treated with exosomes derived from ovarian cancer. Exosomes from untreated ovarian cancer cells, both SKOV-3 and TOV-21G, contributed to a greater decrease in expression of this gene (compared to exosomes from drug-treated ovarian cancer cells). The available literature data suggest that the lack or decreased expression of p53 protein is associated with a faster disruption of the cell cycle and cell dysfunction [50]. It can be assumed that the action of drugs (α-mangostin and/or cisplatin) on ovarian cancer cells may translate not only into changes in the neoplastic cells themselves, but also into slower changes in newly emerging secondary foci. An additional aspect that should be considered in the context of the observed decrease in *TP53* gene expression is cell chemosensitivity. It is well known that mutations in the *TP53* gene contribute to chemotherapy resistance of neoplastic cells, including ovarian cancer [51]. Hence, it is necessary to conduct further research and analyze the cause of the decrease in *TP53* expression in fibroblasts after treatment with tumor-derived exosomes.

The APC protein is involved in the regulation of the cell cycle in several steps: during the G1/S and G2/M transition and during base excision repair. Inhibition of the APC protein activity leads to unregulated cell progression, which has been extensively studied and described for example in colorectal cancer [52,53,54]. In our experiment, we noted a decrease in the expression of the *APC* gene in fibroblasts after treatment with ovarian cancer-derived exosomes. This may lead to disturbance of the adhesion process and dysregulation of the cell cycle and thus contribute to the faster development of the metastatic focus. The lower decrease in *APC* gene expression in exosome-treated fibroblasts from drug-treated ovarian cancer cells indicates that these drugs may also contribute to the inhibition of the metastatic process at this stage. Another aspect is the fact that the loss of *APC* expression results in greater resistance to chemotherapy by enhancing DNA repair and altering the expression of ATP-dependent binding cassette transporters [55].

The role of the *BRMS1* gene (Breast cancer metastasis suppressor 1) in the metastasis process has been poorly understood so far. The available literature data indicate participation in cell communication, mobility, invasion and survival of cells [56]. *BRMS1* plays the role of a suppressor gene as initially demonstrated in breast cancer cells. Over time, research has expanded to show that it also plays a role in melanoma, non-small cell lung cancer, rectal cancer, and ovarian cancer. Decreased *BRMS1* gene expression correlates with poor prognosis and the development of metastasis [57]. The exact mechanism of action is not yet understood, but it is thought to act on NFκ-B signaling by blocking IκBα [58]. In our study, we observed a decrease in *BRMS1* gene expression in fibroblasts after treatment with exosomes derived from both SKOV-3 and TOV-21G cells. The observed decrease in the expression of this gene was smaller when the exosomes were derived from α-mangostin and/or cisplatin-treated ovarian cancer cells. The studies indicate that the *BRMS1* suppressor gene may play an important role in the inhibition of ovarian cancer metastasis [59]. Further work is needed on the role of *BRMS1* in metastasis. However, our research indicates that drugs such as α-mangostin and cisplatin may limit this process by influencing the expression of this gene.

Ovarian cancer is a complex neoplasm and may show different developmental mechanisms, depending on the subtype. The available studies show that the expression of the *HRAS* gene (V-Ha-ras Harvey rat sarcoma viral oncogene homolog) is strong in ovarian cancer cells. Silencing the expression of this gene leads to increased apoptosis and inhibition of tumor growth [60,61]. In our research, we noted an over 1000-fold increase in the expression of the *HRAS* gene in fibroblasts after the treatment with exosomes derived from untreated ovarian cancer cells. In contrast, after treatment of normal fibroblasts with exosomes from ovarian cancer cells treated with α-mangostin and/or cisplatin, the increase was significantly lower (between 8.41% and 183.73%). The *HRAS* gene belongs to the group of RAS GTPases that play an important role in the development of many types of cancer. The exact mechanisms of action of this gene are not fully understood due to the very wide involvement in various molecular pathways. It is known that an increase in *HRAS* expression is associated with the development of metastatic processes, proliferation, apoptosis and cell differentiation [62,63]. Our results show that α-mangostin and/or cisplatin have an impact not only on the ovarian cancer cells themselves, but also on the microenvironment. Inhibition of the increase in *HRAS* expression in cells targeted for the development of metastatic processes may significantly limit the development of metastatic foci of ovarian cancer.

In the development of metastasis, the ability of cells to grow and proliferate is extremely important. It is a complex process in which many factors play a role. In our research, we have shown that exosomes derived from ovarian cancer cells alter the expression of several genes involved in this process. Importantly, the changes we observed relate to genes playing a role at different stages of cell growth and proliferation. In our experiment in which we treated fibroblasts with exosomes derived from SKOV-3 and TOV-21G cells, we observed a change in the expression of the *MDM2* gene (Mdm2 p53 binding protein homolog, mouse). Many studies indicate the role of MDM2 ubiquitin ligase in *TP53* regulation, also in ovarian cancer. Overexpression of *MDM2* has been confirmed to be associated with *TP53* inactivation [64,65,66]. In our research, we also observed a similar relationship. In exosome-treated fibroblasts from untreated SKOV-3 and TOV-21G cells, we observed the greatest increase in *MDM2* expression, while the largest decrease in *TP53* expression. The observed increase in *MDM2* expression following the treatment of exosomes derived from ovarian cancer cells treated with α-mangostin and/or cisplatin was also associated with a proportional decrease in *TP53* gene expression. Overexpression of *MDM2* via *TP53* expression influences cell development. This knowledge is important in the context of the emergence of new metastatic foci. There are also many studies on the use of substances of natural origin to target the p53-MDM2 pathway. The available studies also indicate a p53-independent (wild-type, mutant or null) effect of natural derivatives on *MDM2*. Considering the wide range of activities of natural derivatives, there are high hopes for their chemotherapeutic application [67]. The potential prognostic role of the *MDM2* gene in the process of metastasis is also indicated [65].

The influence of growth factors and cytokines is very important for cell growth. In our study, we observed an increase in the expression of *VEGFA* and three cytokines (*IL18*, *CCL7* and *CXCL12*) in fibroblasts after treatment with exosomes derived from ovarian cancer cells. The role and significance of the noted increase in *VEGFA* expression in fibroblasts following treatment with exosomes derived from TOV-21G cells was discussed above. In the context of the role that *VEGFA* plays in the growth of neoplastic cells, it is worth mentioning the important non-endothelial role of *VEGF*, which is receiving more and more attention. Intracellularly located *VEGF* is an important molecule responsible for signal transduction that regulates cell growth and proliferation [68].

Our results showed that treatment of fibroblasts with exosomes from ovarian cancer cells also changed the expression of the *IL18* gene. The role of interleukin 18 in cancer development is not fully understood. Depending on the time of exposure to IL18, it either accelerates the growth of metastasis or has an inhibitory effect. Nevertheless, many studies show that the use of IL18 binding protein (IL18BP, a soluble protein that binds to IL18), which will neutralize the effects of this interleukin, may have a positive therapeutic effect on cancer [69]. Our observations showed an increase in *IL18* gene expression in fibroblasts as a result of treatment with exosomes from ovarian cancer cells. For both tested lines, the recorded increase in expression was the highest in the exosomes of untreated SKOV-3 and TOV-21G cells. The physiological role of interleukin 18 in ovarian function is very important. It includes participation in follicle development, ovulation and steroidogenesis [70]. Although the exact role of IL18 has not yet been investigated, studies have shown that the serum levels of IL18 in patients with ovarian cancer were elevated compared to controls. On the other hand, studies indicate that interleukin 18 activity may also be affected by the polymorphism of the IL18 gene [71].

Chemokines are a very large group of cytokines that are involved in various processes. They are responsible for leukocyte migration, angiogenesis, tumor growth, and metastasis. There are four main groups of chemokines [72]. In our studies, we checked the change in the expression of two chemokines that play an important role in the metastasis process. The results indicate significant changes in the expression of both *CCL7* and *CXCL12* in fibroblasts after treatment with exosomes derived from ovarian cancer cells. *CCL7* (MCP-3, Chemokine (C-C motif) ligand 7), also known as monocyte chemotactic protein 3 (MCP3), is physiologically expressed in stromal cells, smooth muscle cells, and keratinocytes, among others. The presence of this chemokine in cancer cells was also confirmed [73]. Our research shows that exosomes from ovarian cancer cells lead to an increase in the expression of *CCL7* in fibroblasts. The observed increase in expression was higher for exosomes derived from untreated SKOV-3 or TOV-21G cells. Considering the strong chemotactic properties of *CCL7* for monocytes, eosinophils, basophils, and dendritic cells, it can be expected that an increase in *CCL7* expression in normal cells will increase leukocyte inflow. The exact role of *CCL7* depending on the cellular origin in the development of metastases was summarized by Liu et al. [73]. It has already been confirmed that CAFs (Cancer-Associated Fibroblasts) are characterized by increased production of *CCL7*, as also shown in our research. The autocrine action of *CCL7* promotes and stimulates faster CAF growth. Our studies indicate that the observed increase in CCL expression is lower when the exosomes are derived from α-mangostin and/or cisplatin-stimulated tumor cells, which indicates that the studied drugs affect not only the cells of the cancer itself but also the microenvironment and the rate of the metastasis process.

The second important chemokine for which we observed significant changes in expression under the influence of exosomes is *CXCL12* (SDF1, Chemokine (C-X-C motif) ligand 12). Chemokines show their activity mainly by affecting the receptors. A specific receptor for the *CXCL12* chemokine is known to be the *CXCR4* receptor (Chemokine (C-X-C motif) receptor 4). In our study, we noted changes in the expression of both the *CXCL12* chemokine and its *CXCR4* receptor in fibroblasts after treatment with exosomes of neoplastic origin. Research shows that cancer tissues contain increased levels of *CXCL12* and *CXCR4*. Increased expression of the *CXCL12* chemokine is associated with an increased adhesive capacity of tumor cells, promotion of angiogenesis, and faster development of metastasis. It also contributes to increasing the survival of cancer cells and their proliferation [74,75]. The increase in *CXCL12* and *CXCR4* expression observed by us confirms the important role of the *CXCL12*/*CXCR4* pathway in tumor development and metastasis. Similar observations were obtained for colorectal cancer, where the expression of *CXCL12* and *CXCR4* was higher in lung metastases than in the primary focus [76]. Moreover, studies in ovarian cancer also indicate an association of the *CXCL12*/*CXCR4* axis with drug resistance. It was also confirmed that the expression of these genes was higher in CAF than in the primary focus of ovarian cells and was the cause of EMT induction [77].

In our research, we also noted a change in the expression of the *SET* gene (SET nuclear oncogene). It encodes a nuclear protein, and its increased expression has been described in various types of cancer (including acute myeloid leukemia and ovarian cancer) [78]. Our results show that exosomes from ovarian cancer cells lead to an increase in *SET* expression in fibroblasts, which is one more example of charge transfer and the influence of neoplastic exosomes on the development of a metastatic focus. The greatest increase in *SET* gene expression in fibroblasts was recorded after treatment with exosomes from untreated SKOV-3 and TOV-21G cells. *SET* is responsible for many molecular processes, including acetylation and transcription of histones, migration, and regulation of the cell cycle. It influences the activity of p53 protein, AP1 protein (transcription factor activator protein-1), and AKT protein kinase (protein kinase B) [79]. The multidirectional mechanism of the *SET* gene actions makes it one of the targets of anti-cancer therapy [80]. We noted a lower increase in *SET* gene expression induced by exosomes from ovarian cancer cells treated with α-mangostin and/or cisplatin. This gives preliminary assumptions that the drugs tested by us also indirectly influence the development of metastases.

A final factor that plays an important role in metastasis is apoptosis. Activation of the cell death process in abnormal cells protects the body against the formation of new metastatic foci [81]. In our research, we showed that exosomes derived from ovarian cancer cells lead to a change in the expression of several genes related to the apoptosis process. These genes include: *CXCR4*, *IL18*, *TIMP3*, and *TP53*. The role of individual genes in the metastasis process has already been discussed in the previous sections of the discussion. The available literature data indicate that the apoptotic effect of individual genes occurs through different signaling pathways. For example, *CXCR4* interacts with the PI3K/Akt/NF-κβ signaling [67]. *IL18* through the paths PI3K/Akt and MEK/ERK 1/2 [82,83]. The changes in gene expression that we observed under the influence of exosomes from drug-treated cancer cells (α-mangostin and cisplatin) lead to smaller changes in the expression of these genes compared to exosomes from untreated ovarian cancer cells. This indicates an important role that drugs play not only in the changes taking place in the primary site but also in the development of metastases.

An important aspect of our research that should be noted is the fact that drugs can affect not only the cells themselves but also the microenvironment (including exosome release). The drugs we tested have different mechanisms of action on cells. They can also influence the release of cells from different populations of exosomes. This is also shown by the results of our research. One quite interesting hypothesis [84] is that especially in cancer cells treated with genotoxic drugs, the number of exosomes transporting genomic DNA (gDNA) increases. This has been linked to instability in the genetic material of cells treated with cytotoxic drugs. Importantly, the instability is reflected, among other things, by an increase in the formation of microRNAs and the release of exosomes containing genomic DNA. This peculiar exosomal “cargo” may exert other, perhaps more potent, effects on cells in the tumor microenvironment. It is noteworthy that the content of gDNA and the number of exosomes are also linked to the ploidy of tumor cells. For cells with an abnormal number of chromosomes, not only does the number of unstable microRNAs increase, but the number of gDNA transporting exosomes released increases. The TOV-21G line is a line with a chromosome number defined as 47 (karyotype 47, XX, +10); by comparison, the SKOV-3 line is a hypodiploid human cell line. The modal chromosome number was 43, occurring in 63.3% of cells. The range was 42 to 45 (according to ATCC data). Perhaps the TOV-21G line is characterized by higher genetic instability with potentially more gDNA-containing exosomes released, and thus may more significantly affect cells treated with exosomes released from this line-unique exosome-mediated bystander effects. Adding to this assumption is the fact that cells of the SKOV-3 line identified as cisplatin-resistant, while those of the TOV-21G line identified as cisplatin-sensitive, it can be assumed that a stronger effect of cisplatin on the genetic material of cells of the TOV-21G line will lead to greater instability of genetic material and “evacuation” of portions of genetic material through the secretion of gDNA-rich exosomes. However, this is a hypothesis whose plausibility needs to be tested.

## 4. Materials and Methods

### 4.1. Cell Culture and Drugs

Two human ovarian cancer lines were used in the research. SKOV-3 cisplatin-resistance human ovarian cancer (adenocarcinoma) cell line from American Type Culture Collection (ATCC^®^ HTB-77™, Manassas, VA, USA) were cultured in RPMI-1640 medium (Lonza, Bazylea, Switzerland) with 2 mM L-glutamine (Lonza, Bazylea, Switzerland) supplemented with 10% FBS (fetal bovine serum, Gibco, Waltham, MA, USA) and gentamycin (50 µg/mL). TOV-21G human ovarian cancer (grade 3, stage III, primary malignant adenocarcinoma; clear cell carcinoma) cell line from the American Type Culture Collection (ATCC^®^ CRL-11730™, Manassas, VA, USA) was grown in the mixture (1:1) of MCDB-105 medium (Biological Industries, Beit-Haemek, Israel), and M-199 Earle’s Salts Base medium (Biological Industries, Beit-Haemek, Israel) supplemented with 15% FBS and gentamycin (50 µg/mL). TOV-21G cells line is a sensitive line for cisplatin.

Additionally, a fibroblast line was used. NHDF Normal Human Dermal Fibroblasts from PromoCell bank (C-12300) were cultured in FBM Medium (Fibroblast Growth Basal Medium, Lonza, Bazylea, Switzerland) supplemented with 10% FBS with the addition of inulin and fibroblast growth factor (FGF) and gentamycin (50 µg/mL). All cell lines were cultivated at 37 °C in a humidified atmosphere of 95% air and 5% CO_2_.

Two chemicals were used in the research. Alpha-Mangostin (α-Mangostin) from Sigma-Aldrich (M3824, Saint Louis, MO, USA) dissolved in methanol at a concentration of 24 mM, and cisplatin from Sigma-Aldrich (P4394, Saint Louis, MO, USA), dissolved in 0.9% NaCl solution at a concentration of 1 mg/mL.

Before isolation of exosomes, cells of all lines were cultured in a medium containing Exosome-Depleted FBS (dFBS) (Gibco).

### 4.2. Cell Viability Assay and Drug Combination Studies

SKOV-3 and TOV-21G cells were treated with α-mangostin and/or cisplatin. The cytotoxicity of the compounds was assessed using the XTT test by determining the IC50 value (half maximal inhibitory concentration). To determine the optimal concentration of drug combinations (α-mangostin and cisplatin), the XTT test was performed, followed by the analysis using the CompuSyn software (ComboSyn, Inc., New York, NY, USA) [18,85,86]. The results of the performed analyzes and the determined concentrations for the subsequent stages of the research are summarized in Table 3 [18].

### 4.3. Treatment of Cells, Isolation, and Measurement of Exosomes

TOV-21 and SKOV-3 cells were grown in 75 cm^3^ flasks (3 × 10^6^ cells for flask) until 85–90% confluence in a medium with dFBS. Based on the cytotoxicity results obtained, cells were treated with α-mangostin and/or cisplatin at selected concentrations (Table 3). In addition, untreated TOV-21G cells, untreated SKOV-3 cells, and fibroblast cells, NHDF, were cultured. Before the treatment, cells were cultured for at least 24 h in an Exosome-Depleted FBS medium. Exosomes were isolated from each culture after treatment and compared to non-treatment control. Isolation of exosomes was performed using Total Exosome Isolation (from cell culture media) reagent (Invitrogen) from 10 mL samples of the medium. The isolation was carried out according to the producer’s instructions. The following exosome isolates were obtained: untreated TOV-21G cells (TOV C), TOV-21G cells treated with α-mangostin (TOV MA), TOV-21G cells treated with cisplatin (TOV CIS), TOV-21G cells treated with α-mangostin and cisplatin (TOV MA/CIS), untreated SKOV-3 cells (SKOV C), SKOV-3 cells treated with α-mangostin (SKOV MA), SKOV-3 cells treated with cisplatin (SKOV CIS), SKOV-3 cells treated with α-mangostin and cisplatin (SKOV MA/CIS). Additionally, as a control, exosomes were isolated from untreated NHDF fibroblast cells (NHDF C).

Total protein expression in exosome samples was estimated using a DS-11 series spectrophotometer/fluorometer (DeNovix, Wilmington, DE, USA). The samples’ size, quantity, and purity were measured by the NTA technique (Nanoparticle Tracking Analysis). The NTA measurement technique uses the Brownian motion of particles. Measurement was performed using the Malvern Panalytical NanoSight NS300 instrument (Malvern, UK). The obtained results were statistically analyzed using NTA 3.2 Dev Build 3.2.16 software (Malvern, UK) [18]. Treatment of cells with drugs and isolation of exosomes was performed in triplicate, and each isolated exosome sample was analyzed with a spectrophotometer and NTA technique.

### 4.4. Treatment of NHDF Cells by Exosomes and RNA Isolation

NHDF fibroblasts were cultured in a 6-well plate (1.2 × 106 cells/well) and grown in a dFBS medium. Cells were treated with the obtained exosome isolates (100 ng) for 24 h. Total RNA was then isolated from each sample using the ZR-Duet™ DNA/RNA MiniPrep (Zymo Research, Irvine, CA, USA) according to the manufacturer’s instructions. Controls consisted of untreated NHDF cells. The samples were prepared in triplicate.

### 4.5. qRT-PCR-Based Microarray Assay for the Evaluation of Genes Involved in Metastasis

The change in the expression of genes involved in the process of neoplastic metastasis was assessed using the Human Tumor Metastasis PCR Array (Qiagen, Hilden, Germany). For each sample, Total RNA (3μg) was reverse transcribed to cDNA using the RT2 First Strand Kit (Qiagen, Hilden, Germany), according to the manufacturer’s protocol. Human Tumor Metastasis PCR Array was performed according to the manufacturer’s recommendations using RT2 SYBR^®^ Green qPCR Mastermix (Qiagen, Hilden, Germany). Recommended temperature profile used for all reactions: HotStart DNA Taq Polymerase for 10 min at 95 °C, followed by 40 cycles of 15 s at 95 °C, 1 min at 60 °C, followed by 10 min at 72 °C (Mx 3000p Stratagene, San Diego, CA, USA). The ΔCq value was converted as the difference between the Cq (quantitation cycle) value of the genes of interest and the Cq value of the reference housekeeping genes. Then, the ΔΔCq value was determined for each sample, and finally, the normalized expression level of the target gene was calculated from the formula: 2 − ΔΔCq. qRT-PCR-based microarray assay had as endogenous controls a list of genes: *ACTB* (Actin, beta), *GAPDH* (Glyceraldehyde-3-phosphate dehydrogenase), *HPRT1* (Hypoxanthine phosphoribosyltransferase 1) and *RPLP0* (Ribosomal protein, large, P0).

## 5. Conclusions

In our study, we attempted to evaluate the effect of exosomes released from ovarian cancer cells on altering gene expression in normal human fibroblast cells, with a particular focus on the expression of genes involved in metastasis. We also evaluated whether the application of drugs (α-mangostin and/or cisplatin) alters gene expression (supposedly exosomes) in cells targeted for secondary foci development. The metastasis process is multifactorial. Cancer cells interact not only with other cancer cells, but also with normal cells. This interaction with normal cells, including fibroblasts, is of great importance for metastasis. As mentioned in the introduction, CAF behaves like active fibroblasts which, being in a constant state of activation, affect not only themselves but also the surrounding cells. Under the influence of exosomes derived from ovarian cancer cells, we have noted a change in the expression of many genes involved in tumor metastasis in fibroblasts. Both the exosomes derived from SKOV-3 and TOV-21G cells contributed to the altered expression of genes, in normal fibroblasts, involved in cell adhesion, ECM remodeling, the cell cycle, cell growth and proliferation, and apoptosis. An important observation concerns the fact that changes in gene expression (which showed statistically significant changes) were found to be lesser when the cells were treated with exosomes derived from drug-treated ovarian cancer cells. In most cases, the differences demonstrated were the greatest between the origin of exosomes from untreated tumor cells and cells treated with α-mangostin or cisplatin alone. Exosomes derived from cells treated simultaneously with α-mangostin and cisplatin contributed to a smaller change in the expression of the analyzed genes, in most cases. However, based on the analysis of our results, with caution in making a clear assessment, we can assume that exosomes released from non-drug-treated tumor cells more strongly affected the fibroblast transcriptome inducing such changes in gene expression that can be associated with activation of resting fibroblasts to CAF. These observations are important in terms of developing new therapies targeting not only the cancer cells themselves but also the fibroblasts as part of the tumor microenvironment. Accurate and personalized characterization of CAFs, or reprogramming of CAFs into resting fibroblasts, could become the basis for developing new therapeutic targets. The introduction of naturally derived compounds, such as α-mangostin, into combination therapy may also prove effective in terms of controlled modulation of the tumor microenvironment. The combination of a natural derivative with the currently used chemotherapeutic agent may also contribute to the reduce the side effects of chemotherapy. The mechanism of action of both α-mangostin and cisplatin on ovarian cancer cells is described in the literature [87,88,89,90,91]. Our research indicates their indirect involvement (through exosomes) in the change in the expression of genes influencing the development of metastases. The changes we have noticed can be a valuable source of information and a starting point for discussions on the introduction of new chemotherapeutic regimens. It is necessary to conduct further research and a detailed analysis of whether the observed changes in the Nevertheless, the results of our research provide much information on the influence of the ovarian cancer microenvironment on the development of metastasis at the molecular level expression of genes involved in metastasis which are reflected at the level of the exosome itself.

## Figures and Tables

**Table 1:** List of genes analyzed using Human Tumor Metastasis PCR Array (Qiagen, Hilden, Germany). Genes which showed a statistically significant significance in the results obtained were marked in red.

Cell Adhesion Molecules	
**Cell Adhesion Molecules**	APC, CD44, CDH1, CDH11, CDH6, FAT1, FXYD5, ITGA7, PNN, SYK, VEGFA
Transmembrane Receptors	CD44 , ITGA7, ITGB3, RPSA
Other Cell Adhesion Molecules	CTNNA1, FN1, MCAM, MGAT5, MTSS1
**Extracellular Matrix (ECM) Molecules**	
Extracellular Matrix (ECM) Proteases	MMP10, MMP11, MMP13, MMP2, MMP3, MMP7, MMP9
Extracellular Matrix (ECM) Protease Inhibitors	TIMP2, TIMP3, TIMP4
Other Extracellular Matrix (ECM) Molecules	COL4A2, HPSE, SERPINE1 (PAI-1)
**Cell Cycle**	
Regulation of the Cell Cycle	APC, BRMS1, CDKN2A (P16INK4A), HRAS, IL1B, KRAS, MTSS1, NF2, NME1 (NM23), PTEN, RB1, TGFB1, TP53 (p53), VEGFA
Cell Cycle Arrest & Checkpoints	CDKN2A (P16INK4A), MYC, RB1, TP53 (p53)
**Cell Growth & Proliferation**	
Negative Regulation of Cell Proliferation	CDKN2A (P16INK4A), CTBP1, GNRH1, IL1B, MDM2, NF2, NME1 (NM23), SSTR2
Positive Regulation of Cell Proliferation	IGF1, IL18, TSHR, VEGFA
Growth Factors and Hormones	GNRH1, HGF, IGF1, TGFB1, VEGFA
Cytokines	CCL7 (MCP-3), CXCL12 (SDF1), IL18, IL1B, TNFSF10 (TRAIL)
Cell Surface Receptors	CXCR2, CXCR4, EPHB2, FGFR4, FLT4 (VEGFR2), KISS1R, MET, NR4A3 (NOR1), PLAUR (UPAR), RORB, SSTR2, TSHR
Other Cell Growth Genes	DENR, EWSR1, HRAS, MYC, SET, SRC, SYK, TRPM1
**Apoptosis**	CXCR4, HTATIP2, IL18, IL1B, TGFB1, TIMP3, TNFSF10 (TRAIL), TP53 (p53)
**Transcription Factors & Regulators**	CHD4, ETV4, EWSR1, HTATIP2, MTA1, MYC, MYCL, NR4A3 (NOR1), RB1, RORB, SMAD2 (MADH2), SMAD4 (MADH4), TCF20, TP53 (p53)
**Other Tumor Metastasis Genes**	CD82, CST7, CTSK, CTSL, KISS1, METAP2, NME4

**Table 2:** Percentage change in gene expression level in fibroblasts under the influence of exosomes of various origins.

Gene/Cell Line	SKOV-3 C	SKOV-3 MA	SKOV-3 CIS	SKOV-3 MA/CIS	TOV-21G C	TOV-21G MA	TOV-21G CIS	TOV-21G MA/CIS
**APC**	**99.39%**	**87.82%**	**93.71%**	**86.27%**	**99.37%**	**78.79%**	**93.60%**	**93.81%**
**CD44**	**829.24%**	30.10%**↑**	**504.20%**	32.91%**↑**	**576.30%**	**283.19%**	**372.95%**	**354.89%**
**CDH1**	**47.03%**	**67.05%**	**26.00%**	**49.53%**	**37.42%**	**53.42%**	**98.99%**	**96.13%**
**FXYD5**	**109.88%**	49.87%**↑**	47.59%**↑**	17.36%↓	361.58%	61.98%**↑**	**133.61%**	**234.56%**
**VEGFA**	53.80%**↑**	27.68%↓	15.72%**↑**	1.15%↓	**114.63%**	**130.15%**	**88.39%**	**86.79%**
**RPSA**	**30.19%**	**83.91%**	**60.77%**	**62.23%**	1.98%**↑**	**67.04%**	**53.47%**	**74.87%**
**MMP10**	**518.37%**	**229.48%**	**137.07%**	**330.45%**	**1051.65%**	81.09%**↑**	**678.33%**	**313.18%**
**MMP2**	**179.59%**	**241.91%**	**254.42%**	47.46%**↑**	**1078.63%**	48.61%**↑**	**107.38%**	82.02%**↑**
**TIMP2**	**246.36%**	**128.27%**	**196.11%**	**137.30%**	**382.59%**	51.47%**↑**	40.25%**↑**	43.06%**↑**
**TIMP3**	19.27%↓	29.60%↓	15.35%↓	29.09%↓	**543.83%**	**119.98%**	**452.10%**	**327.60%**
**BRMS1**	**96.37%**	**78.58%**	**84.43%**	**94.40%**	**91.21%**	**72.59%**	**74.60%**	**83.28%**
**HRAS**	**1590.08%**	14.47%**↑**	**151.61%**	52.19%**↑**	**1176.03%**	**142.21%**	8.41%**↑**	**183.73%**
**SET**	**58.26%**	**27.59%**	**51.94%**	**28.27%**	**188.20%**	**143.78%**	**117.53%**	**142.40%**
**TP53**	**71.28%**	**16.22%**	**27.85%**	**46.03%**	**33.96%**	**46.88%**	**59.00%**	4.31%↓
**MDM2**	**141.38%**	41.05%**↑**	46.42%**↑**	17.24%**↑**	61.11%**↑**	36.23%**↑**	38.84%**↑**	60.42%**↑**
**IL18**	**235.74%**	**72.06%**	**108.94%**	33.00%**↑**	**222.51%**	**149.71%**	**149.64%**	55.51%**↑**
**CCL7**	**252.36%**	68.80%**↑**	**226.99%**	34.13%**↑**	**727.94%**	**216.20%**	**345.70%**	**534.28%**
**CXCL12**	**458.65%**	28.53%**↑**	**122.94%**	**219.99%**	**240.47%**	28.78%**↑**	**168.37%**	**65.49%**
**CXCR4**	**165.75%**	31.19%**↑**	**78.77%**	23.93%**↑**	**92.08%**	30.30%**↑**	22.70%**↑**	68.72%

NHDF cells not treated with exosomes were taken at the level of 100%. The red color shows the genes that showed a statistically significant increase in expression (*p* < 0.05). The red arrow shows the genes that showed a statistically insignificant increase in expression. The green color shows the genes that showed a statistically significant decrease in expression (*p* < 0.05). The green arrow shows the genes that showed a statistically insignificant decrease in expression.

**Table 3:** α-Mangostin and/or cisplatin concentrations used to treat SKOV-3 and TOV-21G cells.

Cell line/Compound	α-Mangostin *	Cisplatin *	α-Mangostin + Cisplatin **
SKOV-3	12.5 µM	55.53 µM	12.5 µM + 25 µM
TOV-21G	29.98 µM	38.78 µM	6.25 µM + 50 µM

* IC25 [µM] value for α-mangostin and cisplatin after treatment of SKOV-3 and TOV-21G cells for 24 h (analysis based on XTT test) ** drug combination concentration: α-mangostin and cisplatin after treatment of SKOV-3 and TOV-21G cells for 24 h (based on XTT test and analysis in CompuSyn software, ComboSyn, Inc., New York, NY, USA).

## Data Availability

Data are available on request from the authors.

## References

[1] Siegel R.L., Miller K.D., Fuchs H.E., Jemal A. (2021). Cancer Statistics, 2021. CA. Cancer J. Clin..

[2] Rosen D.G., Yang G., Liu G., Mercado-Uribe I., Chang B., Xiao X., Zheng J., Xue F.X., Liu J. (2009). Ovarian Cancer: Pathology, Biology, and Disease Models. Front. Biosci..

[3] Siegel R.L., Miller K.D., Jemal A. (2020). Cancer Statistics, 2020. CA. Cancer J. Clin..

[4] Lawrie T.A., Winter-Roach B.A., Heus P., Kitchener H.C. (2015). Adjuvant (Post-Surgery) Chemotherapy for Early Stage Epithelial Ovarian Cancer. Cochrane Database Syst. Rev..

[5] Ray-Coquard I., Pautier P., Pignata S., Pérol D., González-Martín A., Berger R., Fujiwara K., Vergote I., Colombo N., Mäenpää J. (2019). Olaparib plus Bevacizumab as First-Line Maintenance in Ovarian Cancer. N. Engl. J. Med..

[6] Pujade-Lauraine E., Ledermann J.A., Selle F., Gebski V., Penson R.T., Oza A.M., Korach J., Huzarski T., Poveda A., Pignata S. (2017). Olaparib Tablets as Maintenance Therapy in Patients with Platinum-Sensitive, Relapsed Ovarian Cancer and a BRCA1/2 Mutation (SOLO2/ENGOT-Ov21): A Double-Blind, Randomised, Placebo-Controlled, Phase 3 Trial. Lancet. Oncol..

[7] Nur A., Ansori M., Fadholly A., Hayaza S., Joko R., Susilo K., Inayatillah B., Winarni D., Akhmad Husen S. (2020). A Review on Medicinal Propertiesof Mangosteen Faheem Ahamed Related Papers A Review on Medicinal Properties of Mangosteen (*Garcinia Mangostana* L.). J. Pharm. Tech..

[8] Nauman M.C., Johnson J.J. (2022). The Purple Mangosteen (Garcinia Mangostana): Defining the Anticancer Potential of Selected Xanthones. Pharmacol. Res..

[9] Barthes J., Ozcelik H., Hindie M., Hasan A., Engin N., Cell V., Özçelik H., Hindié M., Ndreu-halili A., Vrana N.E. (2014). Cell Microenvironment Engineering and Monitoring for Tissue Engineering and Regenerative Medicine: The Recent Advances. Biomed. Res. Int..

[10] Li H., Fan X., Houghton J. (2007). Tumor Microenvironment: The Role of the Tumor Stroma in Cancer. J. Cell. Biochem..

[11] Wang M., Zhao J., Zhang L., Wei F., Lian Y., Wu Y., Gong Z., Zhang S., Zhou J., Cao K. (2017). Role of Tumor Microenvironment in Tumorigenesis. J. Cancer.

[12] Luo Z., Wang Q., Lau W.B., Lau B., Xu L., Zhao L., Yang H., Feng M., Xuan Y., Yang Y. (2016). Tumor Microenvironment: The Culprit for Ovarian Cancer Metastasis. Cancer Lett..

[13] Giusti I., Di Francesco M., D’Ascenzo S., Palmerini M.G., Macchiarelli G., Carta G., Dolo V. (2018). Ovarian Cancer-Derived Extracellular Vesicles Affect Normal Human Fibroblast Behavior. Cancer Biol. Ther..

[14] Shiga K., Hara M., Nagasaki T., Sato T., Takahashi H., Takeyama H. (2015). Cancer-Associated Fibroblasts: Their Characteristics and Their Roles in Tumor Growth. Cancers.

[15] Mitra A.K., Zillhardt M., Hua Y., Tiwari P., Murmann A.E., Peter M.E., Lengyel E. (2012). MicroRNAs Reprogram Normal Fibroblasts into Cancer-Associated Fibroblasts in Ovarian Cancer. Cancer Discov..

[16] Wang Z., Chen J.-Q., Liu J.-L., Tian L. (2016). Exosomes in Tumor Microenvironment: Novel Transporters and Biomarkers. J. Transl. Med..

[17] Zhang Y., Liu Y., Liu H., Tang W.H. (2019). Exosomes: Biogenesis, Biologic Function and Clinical Potential. Cell Biosci..

[18] Borzdziłowska P., Bednarek I. (2022). The Effect of α-Mangostin and Cisplatin on Ovarian Cancer Cells and the Microenvironment. Biomedicines.

[19] Gaona-Luviano P., Adriana L., Magaña-Pérez K. (2020). Epidemiology of Ovarian Cancer. Chin. Clin. Oncol..

[20] Becker A., Thakur B.K., Weiss J.M., Kim H.S., Ctor Peinado H., Lyden D. (2016). Cancer Cell Perspective Extracellular Vesicles in Cancer: Cell-to-Cell Mediators of Metastasis. Cancer Cells.

[21] Pérez-Rojas J.M., Cruz C., García-López P., Sánchez-González D.J., Martínez-Martínez C.M., Ceballos G., Espinosa M., Meléndez-Zajgla J., Pedraza-Chaverri J. (2009). Renoprotection by α-Mangostin Is Related to the Attenuation in Renal Oxidative/Nitrosative Stress Induced by Cisplatin Nephrotoxicity. Free Radic Res..

[22] Sánchez-Pérez Y., Morales-Bárcenas R., García-Cuellar C.M., López-Marure R., Calderon-Oliver M., Pedraza-Chaverri J., Chirino Y.I. (2010). The α-Mangostin Prevention on Cisplatin-Induced Apoptotic Death in LLC-PK1 Cells Is Associated to an Inhibition of ROS Production and P53 Induction. Chem. Biol. Interact..

[23] Nguyen H.L., Hough R., Bernaudo S., Peng C. (2019). Wnt/β-Catenin Signalling in Ovarian Cancer: Insights into Its Hyperactivation and Function in Tumorigenesis. J. Ovarian Res..

[24] Arend R.C., Londoño-Joshi A.I., Straughn J.M., Buchsbaum D.J. (2013). The Wnt/β-Catenin Pathway in Ovarian Cancer: A Review. Gynecol. Oncol..

[25] Narayan S., Roy D. (2003). Role of APC and DNA Mismatch Repair Genes in the Development of Colorectal Cancers. Mol. Cancer.

[26] Sancho-Albero M., Navascués N., Mendoza G., Sebastián V., Arruebo M., Martín-Duque P., Santamaría J. (2019). Exosome Origin Determines Cell Targeting and the Transfer of Therapeutic Nanoparticles towards Target Cells. J. Nanobiotechnol..

[27] Mishra M.N., Chandavarkar V., Sharma R., Bhargava D. (2019). Structure, Function and Role of CD44 in Neoplasia. J. Oral Maxillofac. Pathol..

[28] Shen X., Wang C., Zhu H., Wang Y., Wang X., Cheng X., Ge W., Lu W. (2021). Exosome-Mediated Transfer of CD44 from High-Metastatic Ovarian Cancer Cells Promotes Migration and Invasion of Low-Metastatic Ovarian Cancer Cells. J. Ovarian Res..

[29] Mesrati M.H., Syafruddin S.E., Mohtar M.A., Syahir A. (2021). CD44: A Multifunctional Mediator of Cancer Progression. Biomolecules.

[30] Georgolios A., Eleftheriadou A., Batistatou A., Charalabopoulos K. (2012). Role of the Recently Identified Dysadherin in E-Cadherin Adhesion Molecule Downregulation in Head and Neck Cancer. Med. Oncol..

[31] Ino Y., Gotoh M., Sakamoto M., Tsukagoshi K., Hirohashi S. (2002). Dysadherin, a Cancer-Associated Cell Membrane Glycoprotein, down-Regulates E-Cadherin and Promotes Metastasis. Proc. Natl. Acad. Sci. USA.

[32] Raman P., Purwin T., Pestell R., Tozeren A. (2015). FXYD5 Is a Marker for Poor Prognosis and a Potential Driver for Metastasis in Ovarian Carcinomas. Cancer Inform..

[33] Nam J.S., Hirohashi S., Wakefield L.M. (2007). Dysadherin: A New Player in Cancer Progression. Cancer Lett..

[34] Liu Y.K., Jia Y.J., Liu S.H., Shi H.J., Ma J. (2021). Low Expression of Fxyd5 Reverses the Cisplatin Resistance of Epithelial Ovarian Cancer Cells. Histol. Histopathol..

[35] Tassi R.A., Gambino A., Ardighieri L., Bignotti E., Todeschini P., Romani C., Zanotti L., Bugatti M., Borella F., Katsaros D. (2019). FXYD5 (Dysadherin) Upregulation Predicts Shorter Survival and Reveals Platinum Resistance in High-Grade Serous Ovarian Cancer Patients. Br. J. Cancer.

[36] Carmeliet P. (2005). VEGF as a Key Mediator of Angiogenesis in Cancer. Oncology.

[37] Jiang X., Wang J., Deng X., Xiong F., Zhang S., Gong Z., Li X., Cao K., Deng H., He Y. (2020). The Role of Microenvironment in Tumor Angiogenesis. J. Exp. Clin. Cancer Res..

[38] Claesson-Welsh L., Welsh M. (2013). VEGFA and Tumour Angiogenesis. J. Intern. Med..

[39] Li X., Hu Z., Shi H., Wang C., Lei J., Cheng Y. (2020). Inhibition of VEGFA Increases the Sensitivity of Ovarian Cancer Cells to Chemotherapy by Suppressing VEGFA-Mediated Autophagy. Onco Targets Ther..

[40] Escalona R.M., Kannourakis G., Findlay J.K., Ahmed N. (2022). Expression of TIMPs and MMPs in Ovarian Tumors, Ascites, Ascites-Derived Cells, and Cancer Cell Lines: Characteristic Modulatory Response Before and After Chemotherapy Treatment. Front. Oncol..

[41] Gialeli C., Theocharis A.D., Karamanos N.K. (2011). Roles of Matrix Metalloproteinases in Cancer Progression and Their Pharmacological Targeting. FEBS J..

[42] Al-Alem L., Curry T.E. (2015). Ovarian cancer: Involvement of the matrix metalloproteinases. Reproduction.

[43] Lengyel E. (2010). Ovarian Cancer Development and Metastasis. Am. J. Pathol..

[44] Rodriguez J.A., Orbe J., de Lizarrondo S.M., Calvayrac O., Rodriguez C., Martinez-Gonzalez J., Paramo J.A. (2008). Metalloproteinases and atherothrombosis: MMP-10 mediates vascular remodeling promoted by inflammatory stimuli. Front. Biosci..

[45] Escalona R.M., Bilandzic M., Western P., Kadife E., Kannourakis G., Findlay J.K., Ahmed N. (2020). TIMP-2 Regulates Proliferation, Invasion and STAT3-Mediated Cancer Stem Cell-Dependent Chemoresistance in Ovarian Cancer Cells. BMC Cancer.

[46] Seo D., Li H., Guedez L., Wingfield P., Diaz T., Salloum R., Wei B.Y., Stetler-Stevenson W.G. (2003). TIMP-2 mediated inhibition of angiogenesis: An MMP-independent mechanism. Cell.

[47] Stetler-Stevenson W.G. (2008). The Tumor Microenvironment: Regulation by MMP-Independent Effects of Tissue Inhibitor of Metalloproteinases-2. Cancer Metastasis Rev..

[48] D’Andrilli G., Kumar C., Scambia G., Giordano A. (2004). Cell Cycle Genes in Ovarian Cancer: Steps Toward Earlier Diagnosis and Novel Therapies. Clin. Cancer Res..

[49] Yang-Hartwich Y., Soteras M.G., Lin Z.P., Holmberg J., Sumi N., Craveiro V., Liang M., Romanoff E., Bingham J., Garofalo F. (2015). P53 Protein Aggregation Promotes Platinum Resistance in Ovarian Cancer. Oncogene.

[50] Duffy M.J., Synnott N.C., O’Grady S., Crown J. (2022). Targeting P53 for the Treatment of Cancer. Semin. Cancer Biol..

[51] Tuna M., Ju Z., Yoshihara K., Amos C.I., Tanyi J.L., Mills G.B. (2019). Clinical Relevance of TP53 Hotspot Mutations in High-Grade Serous Ovarian Cancers. Br. J. Cancer.

[52] Noe O., Filipiak L., Royfman R., Campbell A., Lin L., Hamouda D., Stanbery L., Nemunaitis J. (2021). Adenomatous Polyposis Coli in Cancer and Therapeutic Implications. Oncol. Rev..

[53] Sieber O.M., Tomlinson I.P., Lamlum H. (2000). The Adenomatous Polyposis Coli (APC) Tumour Suppressor–Genetics, Function and Disease. Mol. Med. Today.

[54] Wang Y., Azuma Y., Moore D., Osheroff N., Neufeld K.L. (2008). Interaction between Tumor Suppressor Adenomatous Polyposis Coli and Topoisomerase IIα: Implication for the G2/M Transition. Mol. Biol. Cell.

[55] Astarita E.M., Maloney S.M., Hoover C.A., Berkeley B.J., Van Klompenberg M.K., Murlidharan Nair T., Prosperi J.R. (2021). Adenomatous Polyposis Coli Loss Controls Cell Cycle Regulators and Response to Paclitaxel in MDA-MB-157 Metaplastic Breast Cancer Cells. PLoS ONE.

[56] Zimmermann R.C., Welch D.R. (2020). BRMS1: A Multifunctional Signaling Molecule in Metastasis. Cancer Metastasis Rev..

[57] Qiu R., Shi H., Wang S., Leng S., Liu R., Zheng Y., Huang W., Zeng Y., Gao J., Zhang K. (2018). BRMS1 Coordinates with LSD1 and Suppresses Breast Cancer Cell Metastasis. Am. J. Cancer Res..

[58] Ae B.J.M., Frost A.R., King J.A., Dyess D.L., Welch D.R., Samant R.S., Shevde L.A. (2008). Epigenetic Silencing Contributes to the Loss of BRMS1 Expression in Breast Cancer. Clin. Exp. Metastasis.

[59] Zhang S., Lin Q.D., Di W. (2006). Suppression of Human Ovarian Carcinoma Metastasis by the Metastasis-suppressor Gene, BRMS1. Int. J. Gynecol. Cancer.

[60] Yang G., Thompson J.A., Fang B., Liu J. (2003). Silencing of H-Ras Gene Expression by Retrovirus-Mediated SiRNA Decreases Transformation Efficiency and Tumorgrowth in a Model of Human Ovarian Cancer. Oncogene.

[61] Liu J., Yang G., Thompson-Lanza J.A., Glassman A., Hayes K., Patterson A., Marquez R.T., Auersperg N., Yu Y., Hahn W.C. (2004). A Genetically Defined Model for Human Ovarian Cancer. Cancer Res..

[62] Santra T., Herrero A., Rodriguez J., von Kriegsheim A., Iglesias-Martinez L.F., Schwarzl T., Higgins D., Aye T.T., Heck A.J.R., Calvo F. (2019). An Integrated Global Analysis of Compartmentalized HRAS Signaling. Cell Rep..

[63] Lyu H., Li M., Jiang Z., Liu Z., Wang X. (2019). Correlate the TP53 Mutation and the HRAS Mutation with Immune Signatures in Head and Neck Squamous Cell Cancer. Comput. Struct. Biotechnol. J..

[64] Makii C., Oda K., Ikeda Y., Sone K., Hasegawa K., Uehara Y., Nishijima A., Asada K., Koso T., Fukuda T. (2016). MDM2 Is a Potential Therapeutic Target and Prognostic Factor for Ovarian Clear Cell Carcinomas with Wild Type TP53. Oncotarget.

[65] Dogan E., Saygili U., Tuna B., Gol M., Gürel D., Acar B., Koyuncuoǧlu M. (2005). P53 and Mdm2 as Prognostic Indicators in Patients with Epithelial Ovarian Cancer: A Multivariate Analysis. Gynecol. Oncol..

[66] Oliner J.D., Saiki A.Y., Caenepeel S. (2016). The Role of MDM2 Amplification and Overexpression in Tumorigenesis. Cold Spring Harb. Perspect. Med..

[67] Qin J.J., Li X., Hunt C., Wang W., Wang H., Zhang R. (2018). Natural Products Targeting the P53-MDM2 Pathway and Mutant P53: Recent Advances and Implications in Cancer Medicine. Genes Dis..

[68] Wiszniak S., Schwarz Q., Haigh J.J. (2021). Exploring the Intracrine Functions of VEGF-A. Biomolecules.

[69] Karki R., Man S.M., Kanneganti T.D. (2017). Inflammasomes and Cancer. Cancer Immunol. Res..

[70] Medina L., Rabinovich A., Piura B., Dyomin V., Shaco Levy R., Huleihel M. (2014). Expression of IL-18, IL-18 Binding Protein, and Il-18 Receptor by Normal and Cancerous Human Ovarian Tissues: Possible Implication of Il-18 in the Pathogenesis of Ovarian Carcinoma. Mediat. Inflamm..

[71] Bushley A.W., Ferrell R., McDuffie K., Terada K.Y., Carney M.E., Thompson P.J., Wilkens L.R., Tung K.H., Ness R.B., Goodman M.T. (2004). Polymorphisms of Interleukin (IL)-1α, IL-1β, IL-6, IL-10, and IL-18 and the Risk of Ovarian Cancer. Gynecol. Oncol..

[72] Zohny S.F., Fayed S.T. (2010). Clinical Utility of Circulating Matrix Metalloproteinase-7 (MMP-7), CC Chemokine Ligand 18 (CCL18) and CC Chemokine Ligand 11 (CCL11) as Markers for Diagnosis of Epithelial Ovarian Cancer. Med. Oncol..

[73] Liu Y., Cai Y., Liu L., Wu Y., Xiong X. (2018). Crucial Biological Functions of CCL7 in Cancer. PeerJ.

[74] Meng W., Xue S., Chen Y. (2018). The Role of CXCL12 in Tumor Microenvironment. Gene.

[75] Peled A., Klein S., Beider K., Burger J.A., Abraham M. (2018). Role of CXCL12 and CXCR4 in the Pathogenesis of Hematological Malignancies. Cytokine.

[76] Wang M., Yang X., Wei M., Wang Z. (2018). The Role of CXCL12 Axis in Lung Metastasis of Colorectal Cancer. J. Cancer.

[77] Zhang F., Cui J.Y., Gao H.F., Yu H., Gao F.F., Chen J.L., Chen L. (2020). Cancer-Associated Fibroblasts Induce Epithelial-Mesenchymal Transition and Cisplatin Resistance in Ovarian Cancer via CXCL12/CXCR4 Axis. Future Oncol..

[78] Dong L., Zhu J., Wen X., Jiang T., Chen Y. (2014). Involvement of SET in the Wnt Signaling Pathway and the Development of Human Colorectal Cancer. Oncol. Lett..

[79] Cristóbal I., Torrejón B., Rubio J., Santos A., Pedregal M., Caramés C., Zazo S., Luque M., Sanz-Alvarez M., Madoz-Gúrpide J. (2019). Deregulation of SET is Associated with Tumor Progression and Predicts Adverse Outcome in Patients with Early-Stage Colorectal Cancer. J. Clin. Med..

[80] Hung M.-H., Wang C.-Y., Chen Y.-L., Chu P.-Y., Hsiao Y.-J., Tai W.-T., Chao T.-T., Yu H.-C., Shiau C.-W., Chen K.-F. (2016). SET Antagonist Enhances the Chemosensitivity of Non-Small Cell Lung Cancer Cells by Reactivating Protein Phosphatase 2A. Oncotarget.

[81] Su Z., Yang Z., Xu Y., Chen Y., Yu Q. (2015). Apoptosis, Autophagy, Necroptosis, and Cancer Metastasis. Mol. Cancer.

[82] Hirata J.I., Kotani J., Aoyama M., Kashiwamura S.I., Ueda H., Kuroda Y., Usami M., Okamura H., Marukawa S. (2008). A Role for IL-18 in Human Neutrophil Apoptosis. Shock.

[83] Asakawa M., Kono H., Amemiya H., Matsuda M., Suzuki T., Maki A., Fujii H. (2006). Role of Interleukin-18 and Its Receptor in Hepatocellular Carcinoma Associated with Hepatitis C Virus Infection. Int. J. Cancer.

[84] Villar-Prados A., Oliphint P.A., Zhang J., Song X., De Hoff P., Morey R., Liu J., Roszik J., Clise-Dwyer K., Burks J.K. (2019). Mechanisms of nuclear content loading to exosomes. Sci. Adv..

[85] Chou T.-C. (2015). Drug Combination Studies and Their Synergy Quantification Using the Chou-Talalay Method. Cancer Res..

[86] Bijnsdorp I.V., Giovannetti E., Peters G.J. (2011). Analysis of Drug Interactions. Methods Mol. Biol..

[87] Mikuła-Pietrasik J., Witucka A., Pakuła M., Uruski P., Begier-Krasin´ ska B., Niklas A., Tykarski A., Książek K. (2019). Comprehensive review on how platinum- and taxane-based chemotherapy of ovarian cancer affects biology of normal cells. Cell. Mol. Life Sci..

[88] Ovalle-Magallanes B., Eugenio-Pérez D., Pedraza-Chaverri J. (2017). Medicinal properties of mangosteen (*Garcinia mangostana* L.): A comprehensive update. Food Chem. Toxicol..

[89] Watanapokasin R., Jarinthanan F., Nakamura Y., Sawasjirakij N., Jaratrungtawee A., Suksamrarn S. (2011). Effects of α-Mangostin on Apoptosis Induction of Human Colon Cancer. World J. Gastroenterol..

[90] Nakagawa Y., Iinuma M., Naoe T., Nozawa Y., Akao Y. (2007). Characterized mechanism of α-mangostin-induced cell death: Caspaseindependent apoptosis with release of endonuclease-G from mitochondria and increased miR-143 expression in human colorectal cancer DLD-1 cells. Bioorg. Med. Chem..

[91] Aldossary S.A. (2019). Review on Pharmacology of Cisplatin: Clinical Use, Toxicity and Mechanism of Resistance of Cisplatin. Biomed. Pharmacol. J..

